# Should we synthesize more than we need: impact of synthetic data generation for high-dimensional cross-sectional medical data

**DOI:** 10.1093/jamia/ocaf169

**Published:** 2025-10-10

**Authors:** Lisa Pilgram, Samer El Kababji, Dan Liu, Khaled El Emam

**Affiliations:** School of Epidemiology and Public Health, University of Ottawa, Ottawa, Ontario, K1H 8M5, Canada; Children’s Hospital of Eastern Ontario Research Institute, Children’s Hospital of Eastern Ontario, Ottawa, Ontario, K1H 8L1, Canada; Department of Nephrology and Medical Intensive Care, Charité – Universitätsmedizin Berlin, Berlin, 10117, Germany; Children’s Hospital of Eastern Ontario Research Institute, Children’s Hospital of Eastern Ontario, Ottawa, Ontario, K1H 8L1, Canada; School of Epidemiology and Public Health, University of Ottawa, Ottawa, Ontario, K1H 8M5, Canada; Children’s Hospital of Eastern Ontario Research Institute, Children’s Hospital of Eastern Ontario, Ottawa, Ontario, K1H 8L1, Canada; School of Epidemiology and Public Health, University of Ottawa, Ottawa, Ontario, K1H 8M5, Canada; Children’s Hospital of Eastern Ontario Research Institute, Children’s Hospital of Eastern Ontario, Ottawa, Ontario, K1H 8L1, Canada

**Keywords:** data sharing, synthetic data, artificial intelligence, patient privacy, health data platforms

## Abstract

**Objective:**

In medical research and education, generative artificial intelligence/machine learning (AI/ML) models to synthesize artificial medical data can enable the sharing of high-quality data while preserving the privacy of patients. Given that such data is often high-dimensional, a relevant consideration is whether to synthesize the entire dataset when only a task-relevant subset is needed. This study evaluates how the number of variables in training impacts fidelity, utility, and privacy of the synthetic data (SD).

**Material and Methods:**

We used 12 cross-sectional medical datasets, defined a downstream task with corresponding *core* variables, and derived 6354 variants by adding *adjunct* variables to the *core.* SD was generated using 7 different generative models and evaluated for fidelity, downstream utility, and privacy. Mixed-effect models were used to assess the effect of *adjunct* variables on the respective evaluation metric, accounting for the medical dataset as a random component.

**Results:**

Fidelity was unaffected by the number of *adjunct* variables in 5/7 SDG models. Similarly, downstream utility remained stable in 6/7 (predictive task) and 5/7 (inferential task) SDG models. Where significant effects were observed, they were minimal, resulting, for example, in a 0.05 decrease in Area under the Receiver Operating Characteristic curve (AUROC) when adding 120 variables. Privacy was not impacted by the number of *adjunct* variables.

**Discussion:**

Our findings show that fidelity, utility, and privacy are preserved when generating a more comprehensive medical dataset than the task-relevant subset.

**Conclusion:**

Our findings support a cost-effective, utility, and privacy-preserving way of implementing SDG into medical research and education.

## Background and significance

One of the main use cases for synthetic data generation (SDG) in medical research and education is to enable the sharing of high-quality data while preserving the privacy of data subjects.^1^ SDG is a process whereby generative models create new data from the input data they were trained on. SDG can also be based on distributions known a priori and informed by background knowledge, published summary statistics, or published risk calculations.^2–6^ In this paper, however, we focus on an SDG that involves an individual-level training dataset with personal health information.

For high-dimensional data, a recurring question is whether it is better to synthesize the entire dataset or only a subset when there is some a priori knowledge about the downstream analytic use of the synthetic data. While the source databases may have a large number of variables, clinical prediction models have a modest number of predictors, with one review reporting a median number of 16 predictors (IQR: 12-26),^7^ 24 (IQR: 13-112),^8^ and another reporting that the median candidate predictors are 38 with 10 of these in the final models.^9^ We provide 2 example use cases to illustrate the problem being addressed.

In medical data science and machine learning (ML) courses, there is a lack of realistic datasets to teach with and for the students to work with for assignments and projects. SDG can be used to generate privacy-preserving and useful datasets for such education scenarios.^10^ However, if a dataset has 100 variables but an assignment would require only a subset of 20 variables, should the entire dataset or the subset be synthesized? The entire dataset would allow the students to perform more exploratory analysis in a more realistic scenario whereby the variable selection has not already been performed. On the other hand, will synthesizing the full dataset have a negative impact on the data privacy and utility?

Similar considerations apply to Electronic Health Records (EHR) that hold great potential for health research as an increasing number of health care providers adopt these systems.^11^ Initiatives like the All of Us Research Program have leveraged EHR alongside additional health information to create a comprehensive health research platform.^12^ Such health research platforms are also increasingly common in other countries, such as the UK Biobank that provides researchers with access to a large-scale biomedical database of the UK population^13^ or the German National Pandemic Cohort Network, a nationwide data and bio sample collection of COVID-19 patients and controls.^14^ These platforms have a large dataset from which subsets are shared with researchers to answer their specified research questions. The question is whether the full high-dimensional dataset should be synthesized in anticipation of data requests or whether should SDG be performed only when specific cohorts are explicitly requested.

**Figure 1.**
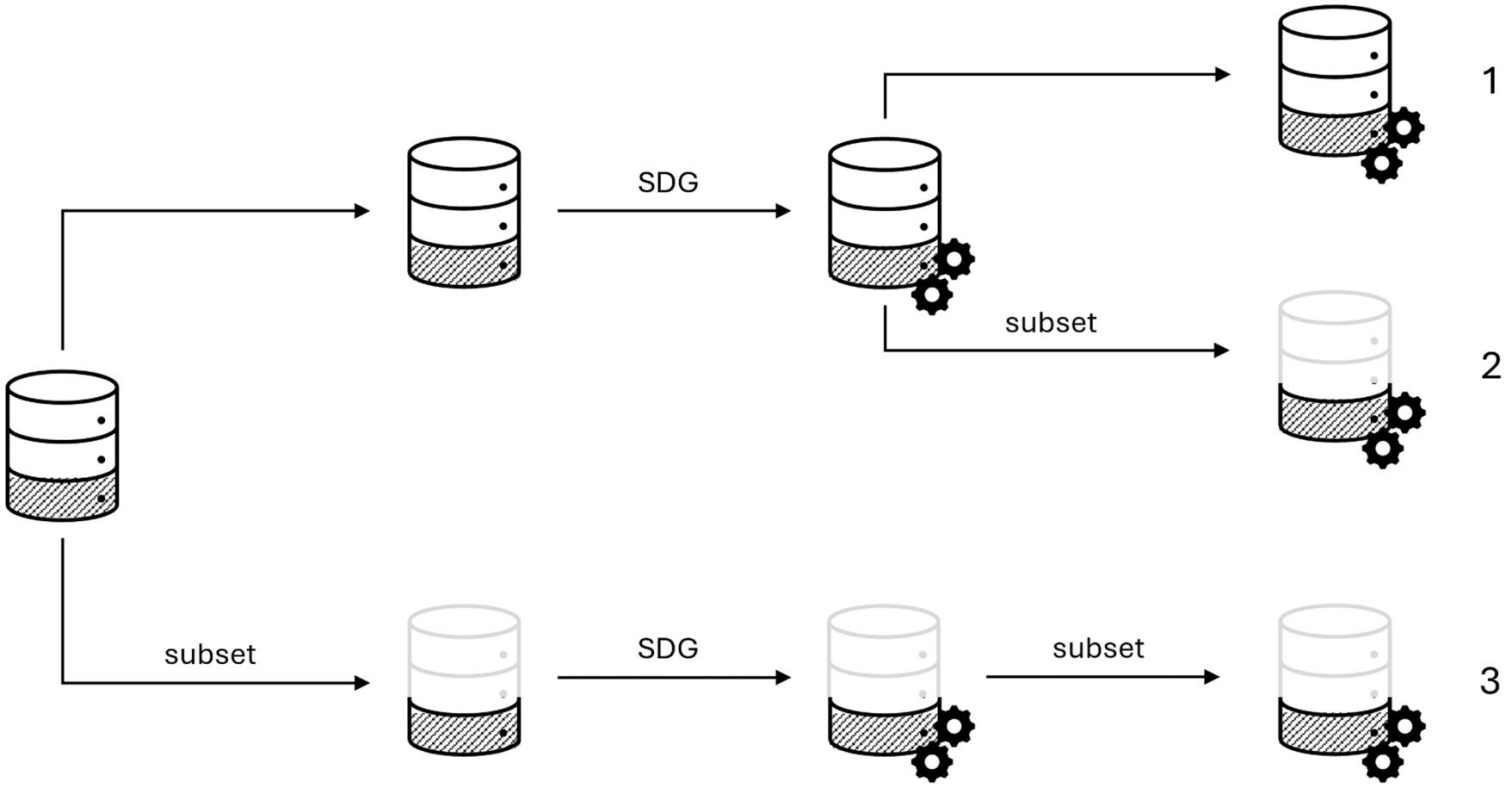
Strategies for Synthetic Data Sharing. Even if the subset of a downstream task is known *a priori* (subset of interest is hatched in the figure), the entire dataset can be used in SDG and the entire synthetic dataset also be released (1); the entire dataset can be used in SDG and the synthetic subset be released (2) or the respective data subset can be used as a training dataset in SDG (3). The decision on the best strategy requires knowledge on the impact of variables on privacy and utility of the synthetic data.

From a practical standpoint, there are 3 strategies for SDG and synthetic data sharing in these examples (see Figure 1).

**Figure 2.**
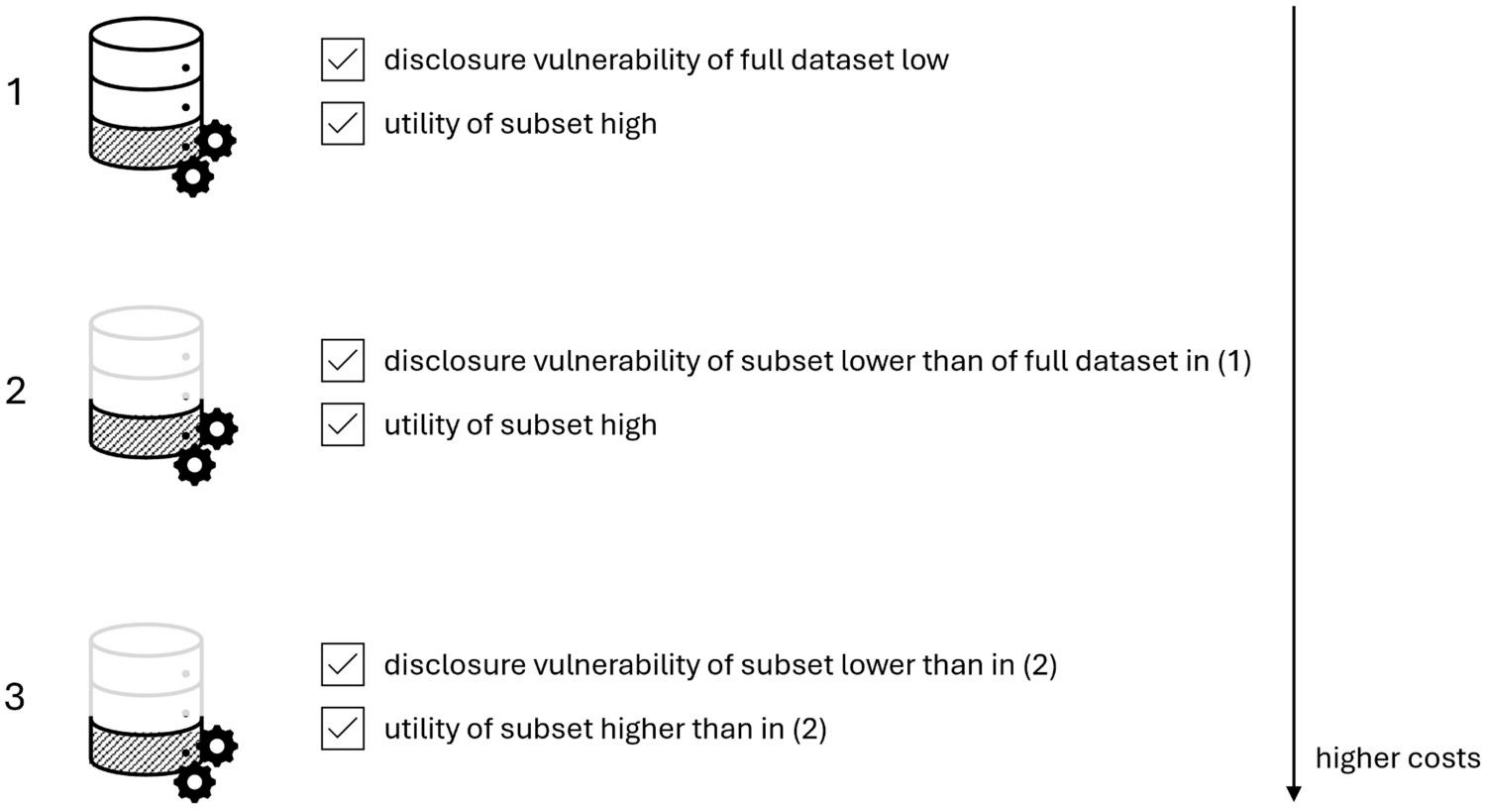
The considerations along the privacy, utility, and cost dimensions for the 3 strategies. The subset of interest is hatched in the figure. The numbers correspond to the release strategies as outlined in Figure 1.

The first strategy is the use of the entire dataset for SDG, and then sharing the entire synthetic dataset. The second one is to disclose the synthetic subset only (ie, the minimum amount of required information for the downstream task) but still use the entire dataset for SDG. And the third strategy is to use the subset for SDG training and to share the resulting synthetic subset. The hypothesized privacy and utility considerations for these 3 strategies, alongside their actual costs, are summarized in Figure 2.

In traditional statistical disclosure control methods, it is well-known that disclosure risk increases the more variables are released.^15–17^ Preserving privacy in high-dimensional datasets is consequently at the cost of utility. A common solution in statistics offices is the release of subsets with varying variables, allowing researchers to select the most relevant one to their research question while maintaining the highest utility possible.^18^ This reflects the general assumption in statistical disclosure control: the more variables, the higher the privacy risk, making it harder to balance the privacy-utility trade-off.

In Ref.^19^, the authors present an improvement in the performance of an SDG model that prioritizes the information from a subset that is relevant to the downstream task over additional variables. In this case, the additional variables may have acted as noise, resulting in the degradation of SDG. Additional variables, however, can also be related to the subset, in particular for medical data, where many variables can be correlated with each other, thereby enabling the generative model to better capture the underlying data distribution (ie, signal). This is very likely dependent on the generative model, as some may be more affected by the signal-to-noise ratio than others. Alongside these utility considerations, we must also account for privacy. Increasing the number of variables could negatively impact privacy, as has been stated in traditional statistical disclosure control. This is reflected in the hypothesized privacy considerations in Figure 2.

There is also the practical consideration of costs, whereby strategies 1 and 2 would be more cost-effective for a data custodian as they involve performing SDG only once for a static database, whereas strategy 3 requires the retention of SDG technology and expertise since SDG is required every time a dataset is requested and disclosed.

The impact of high-dimensional versus low-dimensional datasets on privacy and utility in SDG remains an open question. This study aims at providing evidence for selecting the most appropriate strategy by examining the impact of the number of additional variables on utility and privacy when generating synthetic medical data. We focus here on high-dimensional cross-sectional data and not on longitudinal or repeated measures data. While generating longitudinal data is also an important challenge, it requires distinct SDG modeling approaches and evaluation practices, which are beyond the scope of the current study.

## Materials and methods

**Figure 3.**
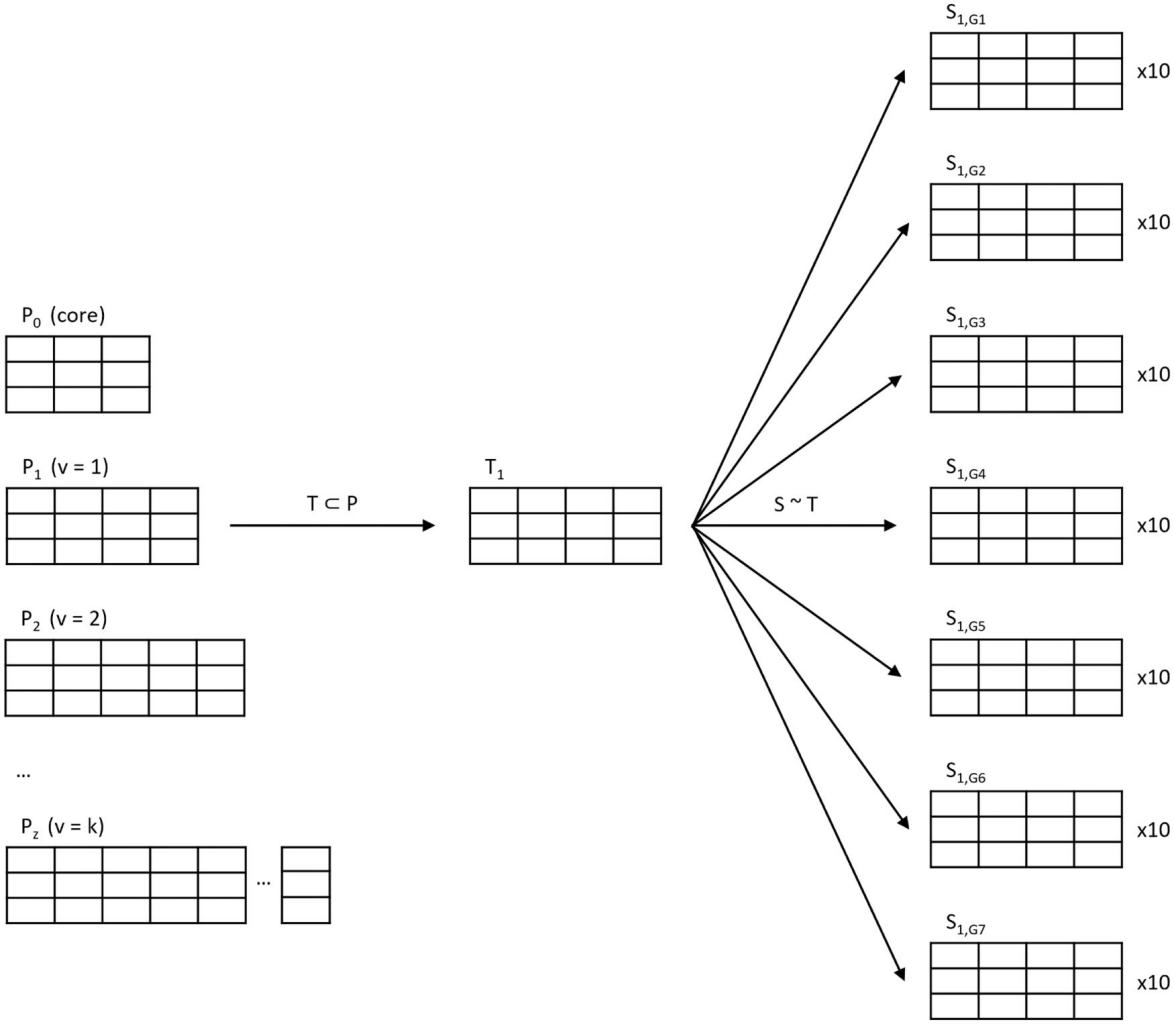
The training datasets for synthetic data generation with an increasing number of variables. Per medical dataset, *core* variables were defined, and *z* variants were created (P_1_-P_z_) by adding an increasing number of *adjunct* variables to the *core* ones. The training dataset (subset T ⊂ P) contained the same random sample of 10 000 records across all population variants. Seven different SDG models (G1-G7) were trained on each training dataset and 10 synthetic datasets (S_1—_S_10_) generated. ⊂: proper subset (subset of randomly drawn or selected records), ∼: SDG, P: Population, S: Synthetic dataset, T: Training dataset.

To analyze the impact of the number of variables in medical data, we defined *core* variables for a downstream task in 12 different medical datasets. We then added an increasing number of variables, trained an SDG model, and evaluated the corresponding *core* synthetic dataset. Seven different generative models were considered: Sequential Decision Trees (ST), Bayesian Networks (BN), Adversarial Random Forests (ARF), Conditional Generative Adversarial Network (CTGAN), Tabular Variational Autoencoder (TVAE), Robust TVAE (RTVAE), and Normalizing Flows (NFlow). The overall workflow included the following steps for each of the 12 medical datasets (see also Figure 3):

1. Definition of *core* variables based on a downstream task
2. Adding *adjunct* variables to the *core* variables, thereby creating so-called *variants* of the medical dataset
3. Sampling the same training records for each *variant* to train an SDG model and generating 10 synthetic datasets of the same size per trained SDG model
4. Reducing each synthetic dataset to its *core* variables and evaluating fidelity (ie, broad utility) and downstream utility
5. Measuring privacy, more precisely membership disclosure vulnerability, in both, the synthetic *core* dataset and the one with *core* and *adjunct* variables

Each step will be described in detail in the following sections:

### Definition of *core* variables

Our research was based on high-dimensional medical datasets (see Table 1). These datasets cover a wide range of typical characteristics (eg, class imbalance, missing values) that are encountered when working with health data.^20^^,^^21^ For each of them, we specified a binary classification task (see online Appendix A) and defined the variables that would be required to perform this task as *core* variables (ie, the predictors and the outcome variables). The imbalance in the outcome variable between majority and minority class ranged from 51% and 49% (ie, approximately balanced) to 99% and 1% (highly imbalanced). The downstream task itself was defined a priori based on domain expertise, related literature, and consultations with the data providers. The datasets with all records but only *core* variables are referred to as *core* populations.

**Table 1.** Medical datasets and variants.

Dataset	Description	**# *core*** $v_{0}$	**pool size** m	# variants
Better Outcomes Registry & Network (BORN)	A dataset that collects data about pregnancy, birth, and childhood in the province of Ontario	20	101	700
California Hospital Discharges 2008 (California)	A dataset that collects the hospital discharge information from the California for 2007	15	387	601
Canadian Community Health Survey (CCHS)	Survey data across multiple years that gathers health information for the Canadian population	13	121	723
Canadian COVID-19 (COVID-19)	A dataset that covers COVID-19 health records of Canadians	6	5	32
FDA Adverse Events (FAERS)	A database that contains adverse event and medication error reports submitted to the FDA	9	27	614
Florida Hospital Discharges 2007 (Florida)	A dataset that collects the hospital discharge information from Florida for 2007	10	293	601
Medical Information Mart for Intensive Care III (MIMIC-III)	A dataset that comprises health data associated with intensive care unit admissions	13	4	16
New York Hospital Discharges 2007 (New York)	A dataset that collects the hospital discharge information from New York for 2007	13	317	601
COVID-19 Survival (NEXOID)	A web-based survey dataset concerning COVID-19 collected by the Nexoid company in London, UK	19	36	622
Texas Inpatient Data (TEXAS)	A dataset on discharges from Texas hospitals	10	65	642
Washington State Hospital Discharges 2007 (Washington)	A dataset that collects the hospital discharge information from Washington for 2007	8	349	601
Washington State Hospital Discharges 2008 (Washington 2008)	A dataset that collects the hospital discharge information from Washington for 2008.	17	407	601

Datasets were, if required, transformed to an individual-level (not event-level) format. For the dataset BORN, the individual was the newborn, not the mother. In the case of FAERS, the database was on event level, but we do not expect a large number of duplicate individuals as adverse events are rare in general. *Core*: number of variables defined for the downstream analysis. Pool size: total number of potential *adjunct* variables.

### Creation of population variants

From the *core* population, population variants were created by adding an increasing number of *adjunct* variables. *Adjunct* variables were those variables present in the original high-dimensional datasets but not part of the *core* subset. Depending on the dimensionality of the medical datasets, the maximum number of potential *adjunct* variables (ie, pool size *m*) varied, and this would have resulted in a large combinatorial space. To reduce the computational burden, we therefore drew random samples from this space but varied the number of samples to achieve a balanced representation of the space for each dataset. The sampling strategy is described in detail in online Appendix A. As a result of this sampling approach, the number of variants with a specific number of *adjunct* variables was generally higher when its combinatorial space was large. A lower boundary was introduced so that each specific number of *adjunct* variables was represented at least by 5 variants, except for cases where the number of *adjunct* variables was equal to the pool size. In these cases, only one variant was included as there was no variety in the combinations. Also, when the total number of possible combinations was small, all possible combinations were included. The final number of variants for each dataset is shown in Table 1, and the distribution of the number of variants across different numbers of *adjunct* variables (ie, how many variants were included for each specific number of *adjunct* variables) is illustrated in online Appendix A. In total, we analyzed 6354 variants derived from 12 medical datasets.

To better understand the characteristics of *core* and *adjunct* variables and to help with interpreting our findings, we assessed how much a *core* variable’s information could be explained by another variable in the dataset. The pairwise normalized mutual information (NMI) was analyzed between *core*-to-*core* and *core*-to-*adjunct* variables.^22^ For implementation, the library infotheo was leveraged.^23^ Results are shown in online Appendix A.

### Synthetic data generation

To exclude any effect of varying sample sizes, we fixed the size of the SDG training dataset at 10 000 records. These were randomly selected records for each medical dataset but remained the same across all population variants for that very dataset. This means that while varying the variables during SDG, the number of records remained the same. For each training dataset, 7 different SDG models were trained (see sidebar). Details on their implementation are provided in online Appendix A.

**Table:** 

Sidebar
There are multiple different SDG models that can be leveraged for tabular synthetic data generation in practice.^24^ The 7 SDG models used in this study are briefly summarized in the following.
**Sequential Decision Trees (ST)** create synthetic data by iteratively modeling one variable at a time, conditional on the previously generated variables.^25–27^
**Bayesian Networks (BN)** are based on directed acyclic graphs with nodes representing the variables and arcs representing the dependencies among these variables. Synthetic data is generated by sampling from the conditional distributions.^28^
**Adversarial Random Forests (ARF)** are a tree-based ensemble, inspired by generative adversarial networks. The adversarial game is a min-max game between a generator and a discriminator (ie, the random forests). Synthetic data is created by sampling from the distributions at those leaf nodes of the random forests where real data is more prevalent.
**Generative Adversarial Networks (GAN)** consist of 2 neural networks, a generator and a discriminator,^29^ playing a min-max game. Conditional tabular GAN (CTGAN) are often used for tabular SDG and designed to better handle imbalanced categorical variables.^30^^,^^31^
**Variational Autoencoders (VAE)** leverage neural networks and include 2 steps. The encoding step compresses the real training data into a lower-dimensional latent space; the decoding step generates synthetic data by sampling from this latent space.^32^ Tabular VAE (TVAE) are designed to handle mixed-type tabular data,^31^ and robust TVAE (RTVAE) extend TVAE by incorporating robust loss functions and regularization techniques.^33^
**Normalizing Flows (NFlow)** generate synthetic data using monotonic rational-quadratic splines.^34^ The spline-based transformations allow the model to maintain exact likelihood computation while capturing complex distributions.

Per trained model, 10 synthetic datasets were generated. This is to account for the stochasticity of the SDG process and is consistent with current practice for SDG.^35–38^

### Evaluating *core* fidelity

In a comparative study, various fidelity metrics, namely, Hellinger distance, Wasserstein distance, cluster analysis, propensity mean squared error, and prediction mean squared error, were able to reflect utility derived from the downstream performance.^39^ Among those, the multivariate Hellinger distance and cluster analysis were found to be the best predictors of how well the synthetic data would perform on downstream analytic workloads.^39^ These metrics consider the multivariate distribution rather than marginal distributions only.

We implemented cluster analysis as a fidelity metric.^40^^,^^41^ This metric clusters synthetic and real records and determines the proportion of real records in each cluster. To be consistent across all evaluation metrics, we scaled it to a range between 0 and 1 with 1 being maximum fidelity. The definition of the metric and its implementation are presented in online Appendix A.

### Evaluating downstream utility

As mentioned previously, for all medical datasets, a downstream task was defined. This was a binary classification task (see online Appendix A). Two typical analytics downstream tasks were evaluated: prediction and inference. The corresponding utility metrics are called train-on-synthetic-test-on-real (TSTR) and the replicability of population inferences.

### Train-On-Synthetic-Test-on-real (TSTR)

Prediction performance of the synthetic data was assessed by training the model on the synthetic data and testing it on a real holdout dataset. This approach is called “train on synthetic, test on real” (TSTR).^42^ The holdout datasets were 10 000 random records, disjoint from the training dataset, and fixed across all population variants for one medical dataset. This corresponds to a 50:50 split for training and holdout dataset. Model performance was assessed as Area under the Receiver Operating Characteristic curve (AUROC).^43^ The AUROC was averaged across the 10 synthetic datasets per trained SDG model. We used 2 different classification models for TSTR, a light gradient-boosting machine (LGBM) and a multi-layer perceptron (MLP), to account for more traditional ML and artificial neural network approaches. For LGBM, hyperparameter tuning was conducted via 5-fold-cross-validation and optimized for AUROC.^44^ For MLP, we did not extensively tune hyperparameters but focused on preventing overfitting.^45^ Implementation details, including hyperparameters for the prediction models, can be found in online Appendix A.

### Replicability of inferences

Replicability occurs when the results from an analysis can be repeated using the same analytical methods but different data.^46^ The analytical method in this study was a logistic regression model, and a clinically relevant parameter of interest was defined for each medical dataset (see online Appendix A). The goal was to obtain a similar estimate for this parameter in the synthetic data to the one in the real data. To evaluate the replicability for the estimate, we used agreement metrics that have been used in the past to compare real-world data analysis results against clinical trials^47^ to assess the replicability of psychological studies^48^ and more recently in the context of synthetic data.^49–51^ These compare the estimates and 95% confidence intervals (CIs) of synthetic data against the real data. Four metrics were defined: *estimate agreement*, *decision agreement, standardized difference,* and *confidence interval overlap* (see Table 2).

**Table 2.** Replicability metrics.

Metric	Explanation
Decision agreement	Whether estimates have the same direction and statistical significance as the real data.
Estimate agreement	Whether the synthetic estimates are within the 95% CI of the real data.
Standardized difference	Whether the difference in the estimates is consistent with the null hypothesis of no difference.
CI overlap	The proportion of the overlap between the synthetic and real 95% CI.

For replicability, we first fitted a logistic regression model on the real data and obtained the estimate and its 95% CI of the parameter of interest. We then trained a logistic regression model on each synthetic dataset. As previously mentioned, to account for the stochasticity during SDG, 10 synthetic datasets were generated per SDG model. This introduces additional variance that needs to be accounted for when reporting the 95% CI of the estimate. This can be done via combining rules similar to those used in multiple imputation.^52^^,^^53^ We describe the implemented combining rules in online Appendix A. The combined estimates and 95% CI across the 10 synthetic datasets were then compared against the estimates derived from the corresponding real dataset, as described in Table 2.

### Evaluating disclosure vulnerability

The privacy literature typically refers to 3 privacy disclosure concepts: identity disclosure, membership disclosure, and attribute disclosure.^1^^,^^54–56^ Identity disclosure is when an individual’s identity can be assigned to a record; membership disclosure is when an individual’s membership in the dataset can be inferred; and attribute disclosure is when an individual’s personal information can be inferred from the attributes of a dataset. Given that throughout SDG, the link to a real record is not preserved, identity disclosure should be protected *by design,* and it is challenging to adapt metrics of identity disclosure to synthetic data to account for this inherent process. Consequently, membership and attribute disclosure are the key vulnerabilities to be evaluated in synthetic data.^57^ While membership disclosure vulnerability is commonly assessed in synthetic medical data,^1^^,^^50^^,^^58^^,^^59^ attribute disclosure vulnerability comes with the challenge of separating inference that can occur independent of any data disclosed from privacy violation.^57^^,^^60^ As there is currently no implementation to measure meaningful attribute disclosure vulnerability, we focused on membership disclosure.

We calculate vulnerability in 2 ways: first, considering quasi-identifiers (QIs) only, and second, using the entire record (all variables). The first way is the most relevant since QIs represent the background knowledge of an adversary.^17^^,^^61–68^ We determined QIs for each of the real-world datasets *a priori* in line with published guidelines (see online Appendix A).^17^^,^^69^ The second way is an unrealistic scenario for an adversary, and more importantly, it can give misleading results.^57^ It can, however, help to detect overfitting. The calculation of membership disclosure vulnerability is explained in online Appendix A.

In addition to the impact of *adjunct* variables on the privacy of the *core* synthetic dataset, we were also interested in the privacy of the entire synthetic dataset (*core* and *adjunct* variables). This means, for each synthetic dataset, we calculated vulnerability for the entire dataset and its *core*. This is relevant to deciding for a release strategy as shown in Figure 1.

### Modeling the effect of the number of adjunct variables

In this study, we used 7 different SDG models, of which some have inherently different underlying processes. We considered it, therefore, very likely that the impact of the number of *adjunct* variables would differ between them and estimated the effect of the number of *adjunct* variables for each SDG model separately.

Within each SDG model, there was still high variability since variants from 12 different medical datasets were considered. Each of these datasets came with its own distribution, its own *core* variables, and its dataset-specific downstream task. This variability needed to be accounted for since it affects SDG, but it also results in varying AUROC and parameter estimates.

Mixed-effect models can account for such variability by allowing a random component while assessing the fixed component. We estimated the fixed effect of *adjunct* variables on fidelity, utility, and privacy through such models. The medical dataset was thereby considered as a random effect; the number of *adjunct* variables as a fixed effect, and the respective evaluation metric (fidelity, utility, or privacy) as the outcome. Since the random effect (ie, the medical dataset) may not only affect the deviation from the mean but also the effect of the *adjunct* variables on the metric, it was considered as both a random intercept and a random slope. Linear mixed-effect models were chosen for the cluster metric (ie, fidelity), the TSTR metric (ie, downstream prediction utility), the 95% CI overlap (ie, downstream replicability utility), and the coefficient (or effect estimate) with respective 95% CI reported. Generalized linear mixed-effect models using a binomial logit link function were chosen for the binary replicability metrics (ie, estimate agreement, the decision agreement, and the standardized difference) and the Odds Ratio (OR) with respective 95% confidence interval (CI) reported as effect size. Mixed-effect models were implemented via the R packages lme4 [70] and lmerTest.^71^ The level of significance was chosen to be 0.05. The 95% CI for the fixed effect estimates was calculated using the likelihood profile method. If the likelihood failed to converge, such as when the model was too complex or data were sparse, we used the Wald CI as an approximation.^70^ P values were computed for fixed effects via Satterthwaite approximation for degrees of freedom as described in Kuznetsova et al.^71^

## Results

In this study, we analyzed if and how the number of *adjunct* variables that are not required for the downstream task affected the generation of synthetic medical data. The results of fidelity, utility, and privacy vulnerability are presented in the following subsections:

### Fidelity

Fidelity was assessed for the synthetic subset that was required for the downstream task (ie, *core*), and the fixed effect estimates of the number of *adjunct* variables when modeling fidelity are shown in Table 3 (together with effect estimates for utility and privacy vulnerability). While fidelity does not necessarily imply downstream utility, it can be a relevant reflection and is more often assessed than a (potentially computationally heavy) downstream workload. In most SDG models (5/7), fidelity was not significantly affected by the number of *adjunct* variables. In 2 models, namely, ARF and RTVAE, fidelity decreased with increasing *adjunct* variables. The estimated effect, however, was small (−0.00099, 95% CI [−0.00148, −0.00049]; −0.00021, 95% CI [−0.00030, −0.00012], respectively).

**Table 3.** The estimated fixed effect of the number of adjunct variables on fidelity and downstream utility.

Model	Evaluation metric	Fixed effect	95% CI (lower)	95% CI (upper)	*P*-value
ST	Cluster metric	−0.00002	−0.00012	0.00007	0.64455
	LGBM TSTR	−0.00004	−0.00017	0.00009	0.51251
	MLP TSTR	−0.00095	−0.00281	0.00087	0.31684
	Estimate agreement	1.00975	0.96675	1.05465	0.66215
	Decision agreement	0.99812	0.97566	1.02109	0.87114
	Standardized difference	1.00700	0.97641	1.03855	0.65761
	95% CI overlap	−0.00113	−0.00335	0.00113	0.32649
BN	Cluster metric	−0.00005	−0.00018	0.00007	0.42270
	LGBM TSTR	0.00013	−0.00004	0.00031	0.14865
	**MLP TSTR**	**0.00016**	**0.00005**	**0.00031**	**0.00759**
	**Estimate agreement**	**1.06513**	**1.00495**	**1.12891**	**0.03347**
	**Decision agreement**	**1.03200**	**1.00304**	**1.06181**	**0.03011**
	**Standardized difference**	**1.05762**	**1.00115**	**1.11727**	**0.04538**
	95% CI overlap	0.00245	−0.00116	0.00586	0.18047
ARF	**Cluster metric**	**−0.00099**	**−0.00148**	**−0.00049**	**0.00318**
	LGBM TSTR	−0.00015	−0.00027	−0.00002	0.06590
	MLP TSTR	0.00001	−0.00010	0.00012	0.84924
	Estimate agreement	0.99155	0.88225	1.11439	0.88672
	Decision agreement	0.97664	0.91271	1.04505	0.49381
	Standardized difference	1.03089	0.91199	1.16528	0.62659
	95% CI overlap	0.00001	−0.00263	0.00265	0.99409
CTGAN	Cluster metric	−0.00009	−0.00023	0.00000	0.05982
	LGBM TSTR	−0.00011	−0.00023	−0.00001	0.06272
	MLP TSTR	−0.00008	−0.00021	0.00002	0.10880
	Estimate agreement	0.99351	0.97410	1.00975	0.41039
	Decision agreement	1.00362	0.99694	1.01034	0.28881
	Standardized difference	0.98748	0.97104	1.00420	0.14130
	95% CI overlap	0.00003	−0.00078	0.00080	0.92544
TVAE	Cluster metric	−0.00009	−0.00016	−0.00001	0.06244
	LGBM TSTR	0.00001	−0.00003	0.00004	0.66203
	MLP TSTR	0.00001	−0.00001	0.00004	0.43761
	Estimate agreement	1.00378	0.98756	1.02027	0.64984
	Decision agreement	1.00580	0.98573	1.02627	0.57387
	Standardized difference	1.00645	0.98826	1.02499	0.48953
	95% CI overlap	0.00037	−0.00135	0.00209	0.68165
RTVAE	**Cluster metric**	**−0.00021**	**−0.00030**	**−0.00012**	**0.00489**
	LGBM TSTR	0.00003	−0.00002	0.00008	0.27865
	MLP TSTR	0.00006	0.00000	0.00012	0.11484
	Estimate agreement	1.00022	0.98707	1.01354	0.97431
	Decision agreement	1.00383	0.99557	1.01216	0.36464
	Standardized difference	0.99759	0.98269	1.01272	0.75375
	95% CI overlap	0.00008	−0.00122	0.00138	0.91019
NFlow	Cluster metric	−0.00015	−0.00035	0.00005	0.16943
	**LGBM TSTR**	**−0.00040**	**−0.00057**	**−0.00023**	**0.00159**
	**MLP TSTR**	**−0.00029**	**−0.00045**	**−0.00013**	**0.00963**
	**Estimate agreement**	**0.99030**	**0.98070**	**1.00000**	**0.04989**
	Decision agreement	0.99447	0.98677	1.00223	0.16191
	Standardized difference	0.99561	0.98432	1.00704	0.44999
	95% CI overlap	−0.00062	−0.00148	0.00023	0.18855

Linear mixed effect models were built for the following metrics with regression coefficient as fixed effect estimates: cluster metric, LGBM TSTR, MLP TSTR, 95% CI overlap. Generalized mixed effect models were built for the following metrics with the odds ratio as fixed effect estimates: estimate agreement, decision agreement, and standardized difference. Medical datasets were considered as random effect. Models with a *P*-value < 0.05 are highlighted in bold.

### Downstream utility

Downstream utility was assessed by TSTR (LGBM) and was not affected by the number of *adjunct* variables in most SDG models (6/7). Only NFlow showed a significant decrease in TSTR utility (LGBM) with increasing *adjunct* variables. Again, the estimated effect was very small (−0.0004, 95% CI [−0.00057, −0.00023]). With 120 *adjunct* variables, this would be a decrease of 0.05 in AUROC. When assessing TSTR (MLP), prediction performance remained stable in most SDG models (6/7) when increasing the number of *adjunct* variables. Again, NFlow presented with a significant but very small effect on the AUROC (−0.00029, 95% CI [−0.00045, −0.00013]).

Replicability was measured by 4 metrics. Generalized mixed models were fitted for the binary metrics, estimate agreement, decision agreement, and standardized difference, and the odds ratio (OR) was estimated; linear mixed models for the 95% CI overlap. In most cases (5/7), the number of *adjunct* variables did not impact replicability. In BN, replicability improved with increasing *adjunct* variables (estimate agreement OR 1.06513, 95% CI [1.00495, 1.12891], decision agreement OR 1.03200, 95% CI [1.00304, 1.06181], standardized difference OR 1.05762, 95% CI [1.00115, 1.11727]); the opposite was the case with NFlow (estimate agreement OR 0.99030, 95% CI [0.98070, 1.00000]). Both, again, with small effect sizes.

Even though it is not the focus of this study, it should be noted that utility, independent of *adjunct* variables, varied across the different medical datasets and SDG models. Utility in AI/ML predictive tasks was typically higher than in the replicability of inferences (see detailed evaluation results in online Appendix A).

### Disclosure vulnerability

Membership disclosure vulnerability was assessed for both the synthetic subset (ie, *core*) and the synthetic dataset with *core* and *adjunct* variables. QIs were used to calculate vulnerability. To select the best SDG and disclosure strategy (see Figure 1), it was not only relevant to know whether the privacy of the synthetic subset with *core* variables changed with increasing *adjunct* variables, but also whether it was low in absolute terms and how it related to the synthetic dataset with *core* and *adjunct* variables. Table 4 shows the estimates for the regression coefficients when the outcome was vulnerability of the synthetic dataset with *core* and *adjunct* variables and when the outcome was vulnerability of the *core* synthetic dataset.

**Table 4.** The estimated fixed effect of the number of adjunct variables on membership disclosure vulnerability.

Model	Synthetic data	Fixed effect	95% CI (lower)	95% CI (upper)	*P*-value
ST	*Core + adjunct* dataset	−0.00001	−0.00002	0.00000	0.12852
	*Core* dataset	0.00000	0.00000	0.00000	0.33311
BN	*Core + adjunct* dataset	0.00006	−0.00002	0.00014	0.26597
	*Core* dataset	0.00001	0.00000	0.00001	0.20498
ARF	*Core + adjunct* dataset	−0.00001	−0.00004	0.00002	0.51088
	*Core* dataset	0.00000	0.00000	0.00000	0.37579
CTGAN	*Core + adjunct* dataset	0.00003	−0.00006	0.00012	0.48038
	*Core* dataset	0.00015	−0.00014	0.00044	0.32674
TVAE	*Core + adjunct* dataset	−0.00005	−0.00014	0.00003	0.40096
	*Core* dataset	0.00000	−0.00001	0.00000	0.27248
RTVAE	*Core + adjunct* dataset	−0.00301	−0.00885	0.00283	0.33623
	*Core* dataset	−0.00238	−0.00706	0.00230	0.34319
NFlow	*Core + adjunct* dataset	0.00001	−0.00004	0.00005	0.76401
	*Core* dataset	0.00000	0.00000	0.00000	0.17116

Mixed effect models were fitted for each SDG model. Vulnerability was calculated for the synthetic dataset with *core + adjunct* variables and for the *core* synthetic dataset separately. Medical datasets were considered as random effect.

Across all SDG models, there was no significant trend with increasing the number of *adjunct* variables. All measured values for membership disclosure vulnerability based on QIs were below 0.2, whether it was the synthetic dataset with *core* and *adjunct* variables or the *core* dataset. This is a threshold that has been used in the literature for the relative F1-score.^50^^,^^57^^,^^59^^,^^72^ Considering this threshold, all generated synthetic datasets would be considered as having a low residual risk of membership disclosure. The maximum value in membership disclosure vulnerability across all SDG models and variants was 0.04, measured in several synthetic datasets of CCHS with *core* and *adjunct* variables generated by BN with more than 78 *adjunct* variables. Leading up to this value, a slowly increasing trend (even though not significant) was observed when increasing the number of *adjunct* variables.

We also calculated vulnerability with all variables (instead of QIs) as a potential indicator for overfitting. Again, no significant impact of increasing *adjunct* variables could be identified across all SDG models and again, higher values could be observed in several synthetic datasets of CCHs generated by BN. Across all other SDG models and medical datasets, the vulnerabilities based on all variables were low (see online Appendix A).

## Discussion

### Summary

This study provides evidence for selecting the most appropriate strategy when using SDG to enable medical data sharing in situations where a *core* subset of a more comprehensive dataset would be sufficient to satisfy the downstream task. For these situations, we analyzed the impact of the number of *adjunct* variables when generating synthetic medical data.

Our analysis shows that the downstream utility of the *core* subset remained stable when increasing the number of *adjunct* variables. While there was a small but significant decrease in fidelity for ARF and RTVAE, the reduced fidelity did not translate into reduced downstream utility. This is in line with research showing that fidelity (or broad utility) does not necessarily reflect downstream utility in synthetic data but also more broadly in privacy-enhancing technologies.^19^^,^^73^ Predictive analytical workloads were not significantly affected by the number of *adjunct* variables, or, if they were, with a very small estimated effect that is unlikely to have practical relevance. Replicability exhibited similar results. Consequently, for the SDG models evaluated in this study, *adjunct* variables did not meaningfully (negatively or positively) affect SDG. This suggests that they did neither act as strong signals to enable the model to better capture the distribution, nor did they introduce unmanageable noise that would degrade SDG. One potential explanation for this finding is that the *adjunct* variables were largely statistically independent of the *core* variables and thus not informative. Another explanation is that *adjunct* variables, while correlated with the *core* variables, did not provide substantial new information beyond what was already captured by the *core* variables. In both scenarios, if SDG models were capable of ignoring such uninformative or redundant input, *adjunct* variables would not relevantly compromise the integrity of the synthetic subset. Our analysis of NMI between *core*-to-*core* and *core*-to-*adjunct* variables (see online Appendix A) showed that in some cases, the *core*-to-*core* NMI was stronger, while in others, the *core*-to-*adjunct* NMI was stronger suggesting that both explanations may hold depending on the dataset.

At the same time, for all SDG models, disclosure vulnerability remained low even when the number of *adjunct* variables reached its maximum of 120. The threshold for acceptable membership disclosure vulnerability in the literature has been 0.2^50^^,^^59^^,^^72^ so that the vulnerability in this study would be considered as an acceptable vulnerability for data release. In BN, when synthesizing CCHS, disclosure vulnerabilities of the synthetic versions showed an increasing tendency. While not significant, this could be interpreted as a sign of SDG overfitting, in particular when considered alongside the positive effect of *adjunct* variables on replicability. The finding that adding *adjunct* variables did not increase membership disclosure vulnerability appears counterintuitive from the perspective of traditional statistical disclosure control methods where it is well-known that disclosure risk increases the more variables are released.^15–17^ However, recent evidence from evaluating synthetic data privacy suggests that an adversary may actually be less successful when using a larger number of variables, as this can increase the probability of mismatches between synthetic and an individual’s ground truth values.^57^

### Implications for generating synthetic medical data

The results of this study are encouraging as they suggest freedom in choosing any strategy depicted in Figure 1. More precisely, even if a downstream task is known *a priori* and only requires a subset of a more comprehensive medical dataset, an entire synthetic dataset can still be generated without impacting the utility of the subset. Privacy of the subset versus the entire synthetic dataset does not differ markedly so that a justifiable sharing strategy can be to share the entire synthetic dataset. If a data controller, perhaps due to data minimization principles or intellectual property, prefers to share the synthetic (*core*) subset, they can still do, but instead of repeating SDG upon each request, they can simply select the subset from the entire synthetic dataset.

For our exemplar scenario in education, the results of this study indicate that educators can share larger medical datasets with their students to give them a more realistic picture of data science without an incremental negative impact on utility and privacy. For increasingly common medical research platforms, researchers could access more data than the minimum amount necessary, which can improve subsequent analyses, and data controllers can reduce costs associated with SDG upon request. The expertise and costs associated with SDG can pose barriers for smaller organization looking to implement SDG to share their medical datasets. For them, SDG can then be a one-time effort, which makes the investment more predictable.

It is important to note that fair and responsible data sharing extends beyond fidelity, utility, and privacy considerations. Ethical implications in SDG must be considered as well, and in theory, as the number of variables increases, the data can become more sparse, resulting in SDG model over- or underfitting, potentially increasing biases present in the training data. There is a growing body of work on how to evaluate fairness in AI/ML,^74–78^ but identifying unwanted bias can be challenging in practice. Certain variables, such as age, may be inappropriate for decisions like hiring yet entirely justified for medical diagnoses where age correlates legitimately with health risk.^79^ Also, in synthetic data, there are no established practices to evaluate fairness, which may be one reason why it is often not evaluated at all.^54^^,^^80^ Another broader concern with any data-driven approach is the exposure of sensitive group-level patterns that could be misused to discriminate against minorities.^81^ This can be through the nature of the data itself but also through the purpose behind the data use. All of this highlights that responsible SDG extends beyond quantitative evaluations to include broader ethical oversight and governance frameworks.

### Limitations

This study has some limitations to indicate. First, our study focused on medical data. In general, results are expected to be transferable to other sectors but depending on the domain, more variables may introduce unmanageable noise or act as a strong signal in SDG. We also limited the maximum number of variables at 120 as we believe that most tabular cross-sectional medical datasets lay within this range or may be only slightly above. Precision molecular datasets and multi-omics data can, however, extend far beyond this number encompassing thousands of variables and conclusions may be substantially different as the sparsity of data could become too pronounced so that SDG models eventually degrade. Second, while we observed consistent findings across datasets, including those where the entire combinatory space was covered, it is important to note that the sampling approach was non-systematic by design and the introduction of systematic biases by the sampling strategy cannot be ruled out. We suggest therefore that future research leverages low-discrepancy sampling methods, such as Halton sequences,^82^ to achieve more uniform coverage across the combinatorial space. Third, SDG models were trained on 10 000 records. We did not vary the training dataset size to reduce potential confounding factors for the evaluation. However, smaller datasets may be affected differently by the number of variables. Future research could explore the relevance of dimensionality in SDG of small data. Fourth, privacy was evaluated as membership disclosure vulnerability. Attribute disclosure vulnerability may also be relevant but current metrics have unresolved methodological challenges.^57^ Fifth, this study was primarily based on de-identified datasets. De-identification can, in general, influence SDG and the fidelity, downstream utility and privacy of the resulting synthetic dataset since eg, outlier records may be removed as part of the de-identification process ultimately reducing the noise in the data. In our study, the level of de-identification varied across datasets due to different data sharing contexts and regulatory frameworks, and findings were consistent across these differently de-identified datasets. Also, utility evaluations are not necessarily sensitive to de-identification. While it is therefore unlikely that de-identification has meaningfully impacted the findings in this study, we acknowledge that, more broadly, de-identification can affect the performance of SDG and should be considered when interpreting evaluation results from SDG. And sixth, the SDG models considered include commonly used models for tabular SDG. However, there are more recent approaches that include diffusion models or large language models, which may present with different behavior.

## Supplementary Material

ocaf169_Supplementary_Data

## Data Availability

All original code for our analyses has been deposited under Ref.^83^ as of the date of publication. Access details for each dataset are provided in the online Appendix A, alongside the specified downstream tasks.
