# Supplementary material for: Should we synthesize more than we need: impact of synthetic data generation for high-dimensional cross-sectional medical data
Source: J Am Med Inform Assoc. 2025 Oct 10;32(12):1843–54. doi: 10.1093/jamia/ocaf169 (PMC12646385; doi:10.1093/jamia/ocaf169)
Supplement: ocaf169_Supplementary_Data [file ocaf169_supplementary_data.docx]

**Should We Synthesize More Than We Need: Impact of Synthetic Data Generation for High Dimensional Cross-Sectional Medical Data – Appendix A**

Lisa Pilgram^1,2,3^, MD; Samer El Kababji^2^, PhD; Dan Liu^1,2^, PhD; Khaled El Emam^1,2^, PhD

^1^School of Epidemiology and Public Health, University of Ottawa, Ontario, Canada;

^2^Children’s Hospital of Eastern Ontario Research Institute, Ontario, Canada;

^3^Department of Nephrology and Medical Intensive Care, Charité – Universitätsmedizin Berlin, Berlin, Germany

**TABLE OF CONTENTS**

[DATA AND DOWNSTREAM TASKS 3](#_Toc204697878)

[Better Outcomes Registry & Network (BORN) 3](#_Toc204697879)

[California State Hospital Discharge Data 4](#_Toc204697880)

[Canadian Community Health Survey (CCHS) 4](#_Toc204697881)

[Canadian COVID-19 Data 5](#_Toc204697882)

[FDA Adverse Event Reporting System (FAERS) 6](#_Toc204697883)

[Florida State Hospital Discharge Data 6](#_Toc204697884)

[Medical Information Mart for Intensive Care III (MIMIC-III) 7](#_Toc204697885)

[New York State Hospital Discharge Data 8](#_Toc204697886)

[COVID-19 Survival (NEXOID) 9](#_Toc204697887)

[Texas State Hospital Discharge Data 10](#_Toc204697888)

[Washington State Hospital Discharge Data 2007 10](#_Toc204697889)

[Washington State Hospital Discharge Data 2008 11](#_Toc204697890)

[Outcome Imbalance in Downstream Tasks 12](#_Toc204697891)

[CREATION OF DATA VARIANTS 12](#_Toc204697892)

[SYNTHETIC DATA GENERATION 14](#_Toc204697893)

[Sequential Decision Trees 14](#_Toc204697894)

[Bayesian Networks 14](#_Toc204697895)

[Adversarial Random Forests (ARF) 15](#_Toc204697896)

[Conditional Generative Adversarial Network 15](#_Toc204697897)

[Variational Autoencoder 15](#_Toc204697898)

[Normalizing Flows (NFlow) 15](#_Toc204697899)

[EVALUATION OF SYNTHETIC DATA 16](#_Toc204697900)

[Fidelity: Cluster Metric 16](#_Toc204697901)

[Train-On-Synthetic-Test-On-Real (TSTR): Prediction Models 16](#_Toc204697902)

[Replicability of Inferences: Combining Rules 17](#_Toc204697903)

[Membership Disclosure Vulnerability 18](#_Toc204697904)

[DETAILED SYNTHETIC DATA EVALUATION RESULTS 22](#_Toc204697905)

[Fidelity 22](#_Toc204697906)

[Downstream Utility 26](#_Toc204697907)

[Membership Disclosure Vulnerability 33](#_Toc204697908)

[MUTUAL INFORMATION ANALYSIS ACROSS VARIABLES 43](#_Toc204697909)

[REFERENCES 45](#_Toc204697910)

# DATA AND DOWNSTREAM TASKS

Each medical dataset came with its specific downstream task as indicated in the following. The downstream task ultimately determined the core variables. This means that the variables provided in the following tables (the outcome variables plus its predictors) are identical to the *core* variables as mentioned in the main manuscript. The downstream task itself was defined a priori based on domain expertise, related literature and consultations with the data providers.

## Better Outcomes Registry & Network (BORN)

The BORN collects Ontario’s prescribed perinatal, newborn and child registry with the role of facilitating quality care for families across the province. It can be accessed through a data request at <https://bornontario.ca/en/data/data.aspx>.

Using data from the BORN, birth weight is predicted considering maternal health conditions, drug intake and gestational age [1], [2]. More precisely, the following variables are used in our analysis:

| **Variable** | **Description** | **Data Type** |
| --- | --- | --- |
| *Birth weight* | Birthweight below 2,500 grams | Binary |
| Gestational age | Time from the first day of the mothers last menstrual period until the date of delivery | Categorical |
| Maternal age | Maternal age at birth | Categorical |
| Maternal BMI | Maternal pre-pregnancy BMI | Categorical |
| Parity | Number of previous pregnancies | Categorical |
| Preterm birth | Number of previous preterm births | Categorical |
| Abortions | Number of previous abortions | Categorical |
| Smoking | Maternal smoking status | Binary |
| Alcohol | Alcohol exposure during pregnancy | Binary |
| Prenatal screening | Conduction of prenatal screening | Binary |
| Addiction | Maternal substance use disorder | Binary |
| Anxiety | Maternal anxiety disorder | Binary |
| Depression | Maternal depressive disorder | Binary |
| Diabetes | Maternal diabetes mellitus | Binary |
| Genetics | Maternal genetic condition | Binary |
| Cocaine drug | Cocaine exposure during pregnancy | Binary |
| Hallucinogens drug | Hallucinogen exposure during pregnancy | Binary |
| Opioids drug | Opioid exposure during pregnancy | Binary |

**Table 1.** **Variables for the Downstream Analysis**. The outcome variable is highlighted in italics. Data type gives information how the variable was measured. BMI: body-mass index.

The regression coefficient of maternal smoking was selected as a representative evaluation point when using logistic regression as downstream task.

## California State Hospital Discharge Data

The California dataset contains the patient’s hospital 2008 discharge data from California, State Inpatient Databases (SID), Healthcare Cost and Utilization Project (HCUP), Agency for Healthcare Research and Quality [3], and is available for purchase at <https://hcup-us.ahrq.gov/tech_assist/centdist.jsp>.

Using data from the California Inpatient Database, length of stay is predicted considering patient’s demographics, their principal diagnosis, comorbidities and undertaken procedures [4], [5]. More precisely, the following variables are used in our analysis:

| **Variable** | **Description** | **Data Type** |
| --- | --- | --- |
| *Length of stay* | Length of stay ≥ 3 days | Binary |
| Age | Patient’s age in years | Numerical |
| Gender | Patient’s gender | Binary |
| Race | Patient’s race | Categorical |
| Weekend admission | Admission on a weekend | Binary |
| DRG | Diagnostic Related Group for prospective payment purposes | Categorical |
| ICD-9 | ICD-9 Diagnosis Code for principal diagnosis | Categorical |
| Chronic condition | Chronic condition of principal diagnosis | Binary |
| Body system | Body system affected by a chronic condition | Categorical |
| Procedure class | ICD Procedure Classes (e.g. minor diagnostic) | Categorical |
| Alcohol abuse | Alcohol abuse as comorbidity | Binary |
| Depression | Depressive disorder as comorbidity | Binary |
| Hypertension | Hypertension as comorbidity | Binary |
| Obesity | Obesity as comorbidity | Binary |
| Primary payer | Expected primary payer (e.g., Medicare) | Categorical |

**Table 2**. **Variables for the Downstream Analysis**. The outcome variable is highlighted in italics. Data type gives information how the variable was measured. DRG: Diagnostic Related Group; ICD: International Classification of Disease.

The regression coefficient of obesity was selected as a representative evaluation point when using logistic regression as downstream task.

## Canadian Community Health Survey (CCHS)

The CCHS data are Canadian population-level information concerning health status, health system utilization and health determinants collected by Statistics Canada through telephone survey. The availability of CCHS data is restricted and requires an access request at <https://www150.statcan.gc.ca/n1/pub/82-620-m/2005001/4144189-eng.htm>.

Using data from the CCHS, cardiovascular risk indicated by the CANHEART Health Index is predicted considering patient’s demographics and cardiovascular health factors [6]. More precisely, the following variables are used in our analysis:

| **Variable** | **Description** | **Data Type** |
| --- | --- | --- |
| *Ideal CANHEART Index* | CANHEART Index ≥ 3 indicating an ideal cardiovascular health status | Binary |
| Age | Patient’s age in years | Categorical |
| Gender | Patient’s gender | Binary |
| Education | Patient’s highest level of education | Categorical |
| Marital status | Patient’s marital status | Categorical |
| House income | Total household income from all sources | Categorical |
| Household size | Size of entire household | Categorical |
| Immigration | Whether a patient is an immigrant | Binary |

**Table 3.** **Variables for the Downstream Analysis**. The outcome variable is highlighted in italics. Data type gives information how the variable was measured. The CANHEART Index is calculated from the following heath factors: smoking, obesity, hypertension, diabetes mellitus, physical activity and fruit and vegetable consumption. CANHEART Index: Cardiovascular Health in Ambulatory Care Research Team health index.

The regression coefficient of gender was selected as a representative evaluation point when using logistic regression as downstream task.

## Canadian COVID-19 Data

The COVID-19 dataset collects Canadian health records of COVID-19 gathered by the Public Health Agency of Canada and is available at Esri Canada (<https://resources-covid19canada.hub.arcgis.com/>).

Using data from the Canadian COVID-19 dataset, case status is predicted considering patient’s demographics, geographical factors and the time when the case occurred [7]. More precisely, the following variables are used in our analysis:

| **Variable** | **Description** | **Data Type** |
| --- | --- | --- |
| *Deceased* | Patient’s status | Binary |
| Age | Patient’s age in years | Categorical |
| Gender | Patient’s gender | Binary |
| Date | Date when the case was reported | Datetime |
| Province | Province in Canada | Categorical |
| Exposure | Type of exposure | Categorical |

**Table 4**. **Variables for the Downstream Analysis**. The outcome variable is highlighted in italics. Data type gives information how the variable was measured.

The regression coefficient of date was selected as a representative evaluation point when using logistic regression as downstream task.

## FDA Adverse Event Reporting System (FAERS)

The FAERS is a database comprising the information on adverse events and medication error reports submitted to FDA and can be downloaded at <https://open.fda.gov/data/faers/>.

Using data from the FAERS, death as outcome from an adverse event is predicted considering patient’s demographics, the drug used and its indication [8]. More precisely, the following variables are used in our analysis:

| **Variable** | **Description** | **Data Type** |
| --- | --- | --- |
| *Death* | Patient’s outcome from adverse event | Binary |
| Age | Patient’s age in years | Numeric |
| Gender | Patient’s gender | Categorical |
| Date | Date of the adverse event | Datetime |
| Weight | Patient’s weight | Numeric |
| Drug | Drug used | Categorical |
| Indication | Indication for drug | Categorical |

**Table 5.** **Variables for the Downstream Analysis**. The outcome variable is highlighted in italics. Data type gives information how the variable was measured.

The regression coefficient of age was selected as a representative evaluation point when using logistic regression as downstream task.

## Florida State Hospital Discharge Data

The Florida dataset contains the patient’s hospital 2007 discharge data from Florida, State Inpatient Databases (SID), Healthcare Cost and Utilization Project (HCUP), Agency for Healthcare Research and Quality [3], and is available for purchase at <https://hcup-us.ahrq.gov/tech_assist/centdist.jsp>.

Using data from the Florida Inpatient Database, length of stay is predicted considering patient’s demographics, their principal diagnosis and charges [4], [9]. More precisely, the following variables are used in our analysis:

| **Variable** | **Description** | **Data Type** |
| --- | --- | --- |
| *Length of stay* | Length of stay ≥ 3 days | Binary |
| Age | Patient’s age in years | Numeric |
| Gender | Patient’s gender | Binary |
| Race | Patient’s race | Categorical |
| ZIP | Patient’s zip code or living status (e.g. homeless) | Categorical |
| Admission type | Type of admission (e.g. emergency) | Categorical |
| Weekend admission | Admission on a weekend | Binary |
| DRG | Diagnostic Related Group for prospective payment purposes | Categorical |
| ICD-9 | ICD-9 Diagnosis Code for principal diagnosis | Categorical |
| Primary payer | Expected primary payer (e.g., Medicare) | Categorical |

**Table 6.** **Variables for the Downstream Analysis**. The outcome variable is highlighted in italics. Data type gives information how the variable was measured. DRG: Diagnostic Related Group; ICD: International Classification of Disease.

The regression coefficient of age was selected as a representative evaluation point when using logistic regression as downstream task.

## Medical Information Mart for Intensive Care III (MIMIC-III)

MIMIC-III is a large database that contains deidentified health-related data associated with over forty thousand patients who stayed in critical care units of the Beth Israel Deaconess Medical Center between 2001 and 2012 [10], [11]. The access to the MIMIC database is upon signing a data use agreement with PhysioNet at <https://physionet.org/content/mimiciii/1.4/> [12].

Using data from the MIMIC-III database, readmission is predicted considering patient’s demographics, vitals and laboratory parameters [13]. More precisely, the following variables are used in our analysis:

| **Variable** | **Description** | **Data Type** |
| --- | --- | --- |
| *Readmission* | *Readmission to Intensive Care Unit within 30 days after discharge* | *Binary* |
| Age | Patient’s age in years | Numeric |
| Ethnicity | Patient’s ethnicity group | Categorical |
| Admission type | Admission type (e.g. emergency) | Categorical |
| Heart rate | Heart rate at admission | Numeric |
| Systolic blood pressure | Systolic blood pressure at admission | Numeric |
| Diastolic blood pressure | Diastolic blood pressure at admission | Numeric |
| Respiratory rate | Respiratory rate at admission | Numeric |
| NT-proBNP | First laboratory test for N-terminal prohormone of brain natriuretic peptide after admission to ICU | Numeric |
| Creatinine | First laboratory test for serum creatinine after admission to ICU | Numeric |
| Blood urea nitrogen | First laboratory test for blood urea nitrogen after admission to ICU | Numeric |
| Potassium | First laboratory test for potassium after admission to ICU | Numeric |
| Cholesterol | First laboratory test for cholesterol after admission to ICU | Numeric |

**Table 7.** **Variables for the Downstream Analysis**. The outcome variable is highlighted in italics. Data type gives information how the variable was measured.

The regression coefficient of heart rate was selected as a representative evaluation point when using logistic regression as downstream task.

## New York State Hospital Discharge Data

The New York dataset contains the patient’s hospital 2007 discharge data from New York, State Inpatient Databases (SID), Healthcare Cost and Utilization Project (HCUP), Agency for Healthcare Research and Quality [3], and is available for purchase at <https://hcup-us.ahrq.gov/tech_assist/centdist.jsp>.

Using data from the New York Inpatient Database, length of stay is predicted considering patient’s demographics, their principal diagnosis and charge information [14], [15], [16]. More precisely, the following variables are used in our analysis:

| **Variable** | **Description** | **Data Type** |
| --- | --- | --- |
| *Length of stay* | Length of stay ≥ 3 days | Binary |
| Age | Patient’s age in years | Numerical |
| Gender | Patient’s gender | Binary |
| Race | Patient’s race | Categorical |
| ZIP | Patient’s zip code or living status (e.g. homeless) | Categorical |
| Admission type | Type of admission (e.g. emergency) | Categorical |
| Weekend admission | Admission on a weekend | Binary |
| DRG | Diagnostic Related Group for prospective payment purposes | Categorical |
| ICD-9 | ICD-9 Diagnosis Code for principal diagnosis | Categorical |
| Chronic condition | Chronic condition of principal diagnosis | Binary |
| Body system | Body system affected by a chronic condition | Categorical |
| Procedure class | ICD Procedure Classes (e.g. minor diagnostic) | Categorical |
| Primary payer | Expected primary payer (e.g., Medicare) | Categorical |

**Table 8**. **Variables for the Downstream Analysis**. The outcome variable is highlighted in italics. Data type gives information how the variable was measured. DRG: Diagnostic Related Group; ICD: International Classification of Disease.

The regression coefficient of age was selected as a representative evaluation point when using logistic regression as downstream task.

## COVID-19 Survival (NEXOID)

The COVID-19 survival dataset is a web-based survey data collected by a company called Nexoid in United Kingdom (UK). It is publicly available at <https://www.covid19survivalcalculator.com/en/download>.

Using data from the COVID-19 web-based survey (Nexoid), risk of infection is predicted considering patient’s demographics, their living circumstances, comorbidities and symptoms [17]. More precisely, the following variables are used in our analysis:

| **Variable** | **Description** | **Data Type** |
| --- | --- | --- |
| *Risk of infection* | Risk of infection ≥ 12.56 | Binary |
| Age | Patient’s age in years | Numerical |
| Gender | Patient’s gender | Categorical |
| Race | Patient’s race | Categorical |
| Smoking | Smoking status | Categorical |
| BMI | Body mass index | Numeric |
| House count | Number of household members | Numeric |
| Public transport count | Frequency of public transport | Numeric |
| Nursing home | Residency in a nursing home | Binary |
| COVID-19 symptoms | Occurrence of symptoms | Binary |
| COVID-19 contact | Contact to a COVID-19 positive person | Binary |
| Health worker | Working in healthcare | Binary |
| Asthma | Asthma | Binary |
| Kidney disease | Kidney disease | Binary |
| Liver disease | Liver disease | Binary |
| Heart disease | Heart disease | Binary |
| Lung disease | Lung disease | Binary |
| Diabetes | Diabetes mellitus | Binary |
| Hypertension | Hypertension | Binary |

**Table 9.** **Variables for the Downstream Analysis**. The outcome variable is highlighted in italics. Data type gives information how the variable was measured.

The regression coefficient of gender was selected as a representative evaluation point when using logistic regression as downstream task.

## Texas State Hospital Discharge Data

The Texas dataset contains the patient’s hospital discharge information for the first quarter of 2012 from Texas in the United States [18], and is publicly available at <https://www.dshs.texas.gov/center-health-statistics/chs-data-sets-reports/texas-health-care-information-collection/health-data-researcher-information/texas-inpatient-public-use>.

Using data from the Texas Inpatient Database, length of stay is predicted considering patient’s demographics, their health condition and charge information [19]. More precisely, the following variables are used in our analysis:

| **Variable** | **Description** | **Data Type** |
| --- | --- | --- |
| *Length of stay* | Length of stay ≥ 3 days | Binary |
| Age | Patient’s age in years | Categorical |
| Gender | Patient’s gender | Categorical |
| Race | Patient’s race | Categorical |
| Hispanic ethnicity | Hispanic origin of patient | Binary |
| State | State of patient’s mailing address in the USA | Categorical |
| Weekday of admission | Day of the week when patient was admitted (e.g. Monday) | Categorical |
| Risk mortality | Risk of mortality | Categorical |
| Disease severity | Severity of illness | Categorical |
| DRG | Diagnostic Related Group for prospective payment purposes | Categorical |

**Table 10**. **Variables for the Downstream Analysis**. The outcome variable is highlighted in italics. Data type gives information how the variable was measured. DRG: Diagnostic Related Group.

The regression coefficient of moderate disease severity was selected as a representative evaluation point when using logistic regression as downstream task.

## Washington State Hospital Discharge Data 2007

The Washington dataset contains the patient’s hospital 2007 discharge data from Washington, State Inpatient Databases (SID), Healthcare Cost and Utilization Project (HCUP), Agency for Healthcare Research and Quality [3], and is available for purchase at <https://hcup-us.ahrq.gov/tech_assist/centdist.jsp>.

Using data from the Washington Inpatient Database (2007), length of stay is predicted considering patient’s demographics and their principal diagnosis [20], [21]. More precisely, the following variables are used in our analysis:

| **Variable** | **Description** | **Data Type** |
| --- | --- | --- |
| *Length of stay* | Length of stay ≥ 3 days | Binary |
| Age | Patient’s age in years | Numeric |
| ZIP | Patient’s zip code or living status (e.g. homeless) | Categorical |
| Admission type | Type of admission (e.g. emergency) | Categorical |
| Weekend admission | Admission on a weekend | Binary |
| DRG | Diagnostic Related Group for prospective payment purposes | Categorical |
| ICD-9 | ICD-9 Diagnosis Code for principal diagnosis | Categorical |
| Deceased | Death of patient during inpatient stay | Binary |

**Table 11**. **Variables for the Downstream Analysis**. The outcome variable is highlighted in italics. Data type gives information how the variable was measured. DRG: Diagnostic Related Group; ICD: International Classification of Disease.

The regression coefficient of age was selected as a representative evaluation point when using logistic regression as downstream task.

## Washington State Hospital Discharge Data 2008

The Washington2008 dataset contains the patient’s hospital 2008 discharge data from Washington, State Inpatient Databases (SID), Healthcare Cost and Utilization Project (HCUP), Agency for Healthcare Research and Quality [3], and is available for purchase at <https://hcup-us.ahrq.gov/tech_assist/centdist.jsp>.

Using data from the Washington Inpatient Database (2008), length of stay is predicted considering patient’s demographics, their principal diagnosis, comorbidities and undertaken procedures [21], [22]. More precisely, the following variables are used in our analysis:

| **Variable** | **Description** | **Data Type** |
| --- | --- | --- |
| *Length of stay* | Length of stay ≥ 3 days | Binary |
| Age | Patient’s age in years | Numerical |
| Gender | Patient’s gender | Binary |
| Race | Patient’s race | Categorical |
| ZIP | Patient’s zip code or living status (e.g. homeless) | Categorical |
| Admission type | Type of admission (e.g. emergency) | Categorical |
| Weekend admission | Admission on a weekend | Binary |
| DRG | Diagnostic Related Group for prospective payment purposes | Categorical |
| ICD-9 | ICD-9 Diagnosis Code for principal diagnosis | Categorical |
| Chronic condition | Chronic condition of principal diagnosis | Binary |
| Body system | Body system affected by a chronic condition | Categorical |
| Procedure class | ICD Procedure Classes (e.g. minor diagnostic) | Categorical |
| Alcohol abuse | Alcohol abuse as comorbidity | Binary |
| Depression | Depressive disorder as comorbidity | Binary |
| Hypertension | Hypertension as comorbidity | Binary |
| Obesity | Obesity as comorbidity | Binary |
| Primary payer | Expected primary payer (e.g., Medicare) | Categorical |

**Table 12. Variables for the Downstream Analysis**. The outcome variable is highlighted in italics. Data type gives information how the variable was measured. DRG: Diagnostic Related Group; ICD: International Classification of Disease.

The regression coefficient of obesity was selected as a representative evaluation point when using logistic regression as downstream task.

## Outcome Imbalance in Downstream Tasks

To better understand the generalizability of the findings from the downstream tasks, the imbalance of the binary outcome variable is reported in Table 13. Across datasets, the degree of imbalance varied from approximately balanced (e.g., Washington 51:49) to highly imbalanced (e.g., COVID-19 99:1).

| **Dataset** | **Outcome Imbalance** |
| --- | --- |
| BORN | 93:7 |
| California | 55:46 |
| CCHS | 87:13 |
| COVID-19 | 99:1 |
| FAERS | 90:10 |
| Florida | 60:40 |
| MIMIC-III | 90:10 |
| New York | 61:39 |
| NEXOID | 61:39 |
| TEXAS | 60:40 |
| Washington | 51:49 |
| Washington 2008 | 58:42 |

**Table 13. Outcome Imbalance for the Downstream Analysis**. The imbalance of the binary outcome variable is indicated as percentage of records in each class (majority : minority).

# CREATION OF DATA VARIANTS

From the datasets described in the previous section of the Appendix, variants of varying dimensionality were created by adding an increasing number of *adjunct* variables to a set of *core* variables. *Core* variables were defined by the downstream tasks described in the previous section.

As described in the main manuscript, depending on the dimensionality of the medical datasets, this would result in a large combinatorial space:

Let $v_{0}$ be the number of *core* variables, and $v$the number of *adjunct* variables. Then, the dimensionality of a dataset is defined by $v_{0}+v$. The maximum number of potential *adjunct* variables is referred to as pool size $m$. It varies depending on the medical dataset and determines the total number of potential combinations. For example, with $v=2$ and $m=100$, we could create $\left(\begin{matrix} 100 \\ 2 \end{matrix}\right)=4950$ distinct population variants with different combinations of the two *adjunct* variables added to the *core* variables. As $v$ranges from one to $m$, the space of population variants would grow up to 1.267651 × 10^30^ distinct population variants in this example.

In the following, we describe the sampling strategy to reduce the computational burden while achieving a balanced representation of that space.

The maximum number of *adjunct* variables was limited to 120 in cases where $m>120$, so that the maximum number of *adjunct* variables in this study was defined as $k=\min\left(120,m\right)$. More precisely, we examined $r_{v}$ population variants for each $v$where$v$ranged from 1 to $k$. For each$v$, the number of population variants $r_{v}$ was determined considering all available population variants for that particular $v$:

| $r_{v}=\max\left(5,\text{ }\left(w_{v}\times600\right)\right)$ |  |
| --- | --- |

This means, we made sure to have a minimum number of 5 variants for each $v$and a maximum number of variants based on the weight $w_{v}$ whereby 600 was a constant factor empirically chosen to consider a feasible weighted $r_{v}$ , so that, in total, the number of variants ranged between 600 and 750 per medical dataset. We set $r_{v}=1$ in cases where the number of *adjunct* variables was equal to the pool size (i.e.,$v=m$*)* since, in this case, the number of possible combinations of variables was one. The weight $w_{v}$ for each $v$ ranging from one to $k$ was then calculated as:

| $w_{v}\text{ }=\frac{\log\left(\begin{matrix} m \\ v \end{matrix}\right)}{d}$ |  |
| --- | --- |

with $d$ acting as a normalizing factor considering all combinations among the potential *adjunct* variables:

| $d=\sum_{i=1}^{m} \log\left(\begin{matrix} m \\ i \end{matrix}\right)$ |  |
| --- | --- |

The maximum pool size $m$was dataset specific. For two datasets, namely Canadian COVID-19 (COVID-19) and Medical Information Mart for Intensive Care III (MIMIC-III), $m$ was very small so that all possible combinations could be considered while the number of variants remained relatively small (31 and 15 respectively). The total number of variants for each dataset is presented in the main manuscript and the result of this sampling approach is illustrated in Figure 1.


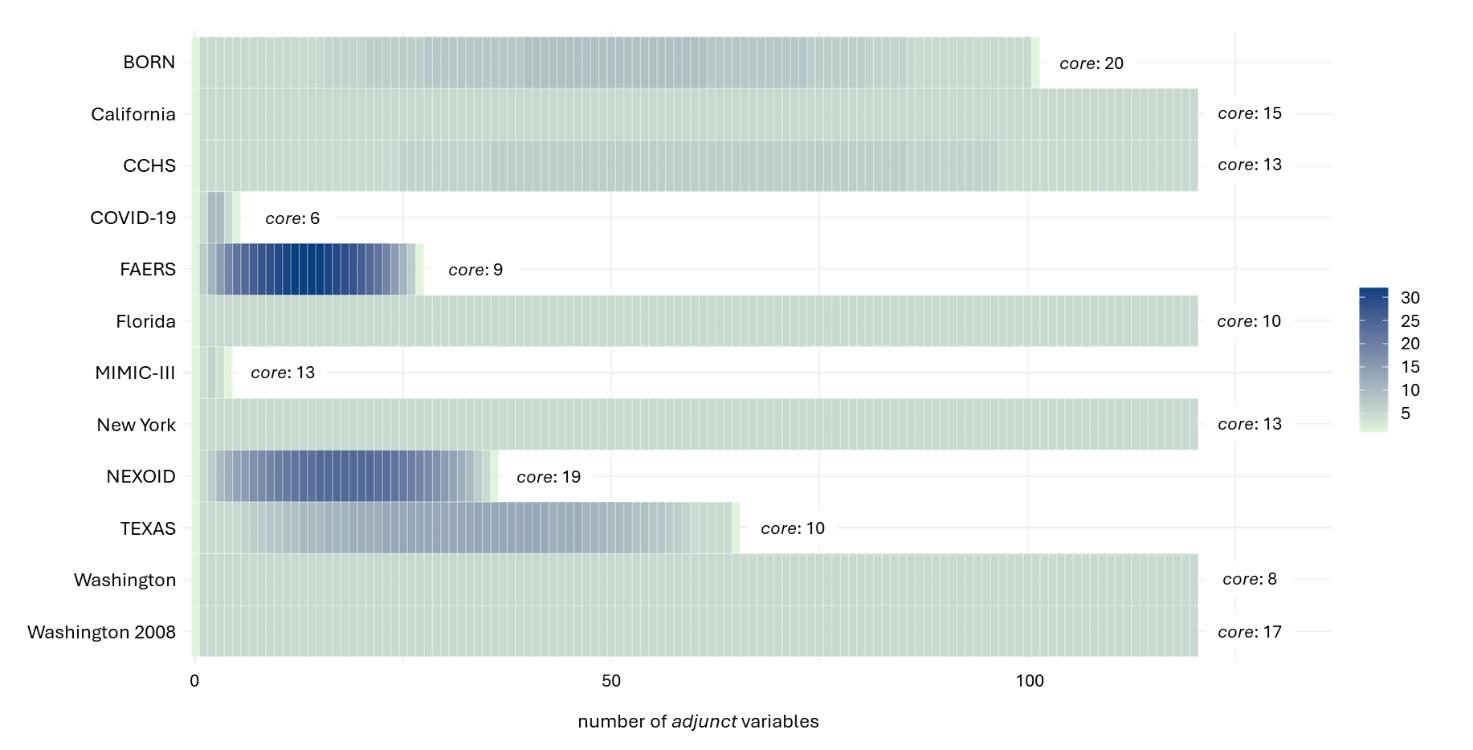


**Figure 1. Number of population variants per number of *adjunct* variables**. The number of population variants for each specific number of *adjunct* variables is color-coded with green indicating a lower and blue a higher number of variants. Note that each number of *adjunct* variables is represented at least by five variants. The number of *core* variables is reported for each dataset (i.e., *core*).

# SYNTHETIC DATA GENERATION

Seven different types of SDG models were considered when evaluating the impact of *adjunct* variables in SDG, namely, sequential decision trees (ST) [23], [24], [25], [26], Bayesian networks (BN) [27], [28], [29], [30], conditional generative adversarial network (CTGAN) [31], variational autoencoders (TVAE and RTVAE) [31], adversarial random forests (ARF) [32], and normalizing flows (NFlow) [33]. They are described in the following.

The first method was implemented using Aetion**®** Generate, a commercial product from Aetion[[1]](#footnote-2), and the last six methods were implemented using an open-sourced Python package Synthcity [34]. Our implementation, the publicly available Python package pysdg [35], provides further pre-processing and post-processing on top of Synthcity.

## Sequential Decision Trees

Similar to using a chaining method for multi-label classification problems, sequential decision trees (ST) generate synthetic data using conditional trees in a sequential fashion [23], [36], [37]. It has been commonly employed in the medical and social science domains for data synthesis [24], [25], [38], [39], [40], [41], [42], [43], [44]. The details of the implementation procedures can be referred to [23].

## Bayesian Networks

Bayesian Networks (BN) are models based on Directed Acyclic Graphs that consist of nodes representing the random variables and arcs representing the dependencies among these variables. To construct the BN model, the first step is to find the optimal network topology, and then to estimate the optimal parameters [27]. Starting with a random initial network structure, the Hill Climb heuristic search is used to find the optimal structure. Then, the conditional probability distributions are estimated using the maximum a posteriori estimator [45]. Once the network structure and the parameters are estimated, we can initialize the nodes with no incoming arcs by sampling from their marginal distributions and predict the rest of the connected variables using the estimated parameters.

## Adversarial Random Forests (ARF)

Adversarial Random Forests is a tree-based density estimator that uses recursive unsupervised random forests [32]. We used the implementation of ARF from Synthcity [46].

Inspired by generative adversarial networks (GANs), ARFs employ a recursive process where trees iteratively learn the structural properties of data by alternating between rounds of data generation and discrimination. This allows the model to gradually refine its understanding of the data distribution. Unlike classic tree-based models, ARFs provide smooth density estimations and can generate fully synthetic data.

## Conditional Generative Adversarial Network

A basic GAN consists of two artificial neural networks (ANNs), a generator and a discriminator [47]. The generator and the discriminatorplay a min-max game. The input to the generator is noise, while its output is synthetic data. The discriminator has two inputs: the real training data and the synthetic data generated by the generator. The output of the discriminator indicates whether its input is real or synthetic. The generator is trained to ‘trick’ the discriminator by generating samples that look real. On the other hand, the discriminator is trained to maximize its discriminatory capability.

Among all the variations of GAN architectures, the conditional tabular GAN (CTGAN) is often used in tabular data synthesis [48]. CTGAN builds on conditional GANs by addressing the multimodal distributions of continuous variables and the highly imbalanced categorical variables [31]. CTGAN solves the first problem by proposing a per-mode normalization technique. For the second problem, each category of a categorical variable serves as the condition passed to the GAN.

## Variational Autoencoder

Variational autoencoders (VAE) use ANNs and involve two steps (encoding and decoding) to generate new samples [49]. First, an encoder is generated to compress input data into a lower-dimensional latent space, in which the data points are represented by distributions. The second step is a decoding process, in which new data samples are reconstructed as output from the latent space. The neural network is optimized by minimizing the reconstruction loss between the output and the input. VAEs are known to generate complex data of various types due to its ability to learn more complex distributions [50]. Many variants have been proposed as an extension of VAE, such as triplet-based VAE [51], conditional VAE [52], and Gaussian VAE [53]. In particular, the tabular VAE (TVAE) was proposed as an adaption of standard VAE to model and generate mixed-type tabular data with a modified loss function [31]. We also used a robust TVAE (RTVAE) as introduced by Akrami et al. [54] and, again, implemented in Synthcity.

## Normalizing Flows (NFlow)

Normalizing Flows are generators based on monotonic rational-quadratic splines [33]. We used the implementation of NFlow from Synthcity [46]. NFlow utilizes monotonic rational-quadratic splines to implement invertible transformations, offering a significant improvement over traditional affine or additive transformations typically used in flow-based models.

# EVALUATION OF SYNTHETIC DATA

## Fidelity: Cluster Metric

We implemented cluster analysis as a fidelity metric [55], [56]. This metric clusters synthetic and real records and determines the proportion of real records in each cluster. To be consistent across all evaluation metrics, we scaled it to a range between 0-1 with 1 being maximum fidelity.

The metric was defined as follows

| $U=1-\left(\frac{1}{G\times(1-c)^{2}}\sum_{j=1}^{G} \left(\frac{n_{j}^{R}}{n_{j}}-c\right)^{2}\right)$ |  |
| --- | --- |

where $G$was the number of clusters, $n_{j}$the number of records in the j-th cluster, $n_{j}^{R}$the number of records from the real dataset in the j-th cluster and $c$a constant defining the relation between the real (i.e.,$n^{R}$) and synthetic dataset sizes (i.e., $n^{S}$).

| $c=\frac{n^{R}}{n^{R}+n^{S}}$ | (1) |
| --- | --- |

Since $n^{R}=n^{S}$in our setting, equation simplified to

| $U=1-\left(\frac{4}{G}\sum_{j=1}^{G} \left(\frac{n_{j}^{R}}{n_{j}}-0.5\right)^{2}\right)$ |  |
| --- | --- |

In k-means clustering, *k* equals the number of clusters which is referred to as $G$in our notation. This was determined for each medical dataset by calculating the bootstrapped gap statistic when clustering the *core* dataset [57]. To conduct clustering, datetime variables were transformed into numerical variables by counting the days since 1900/01/01. Numerical variables were then discretized into 20 bins ranging from the minimum to the maximum value. Discretizing was performed uniformly in corresponding real and synthetic datasets to have matching categories.

## Train-On-Synthetic-Test-On-Real (TSTR): Prediction Models

Prediction performance of the synthetic data was assessed by training the model on the synthetic data and testing it on a real holdout dataset (i.e., TSTR) [58]. The following two prediction models were used to account for a more traditional ML and a recent neural network approach.

Light gradient boosting machines (LGBM) were selected as ML model. This is an ensemble approach and among the most deployed classifiers in ML [59], [60]. Model training was optimized for AUROC using 5-fold-cross-validation [61]. During cross-validation, the following hyperparameters were considered:

| **Hyperparameter** | **Default Value** | **Lower Bound** | **Upper Bound** |
| --- | --- | --- | --- |
| Booster | 1 (gradient boosting decision tree) | 1 (gradient boosting decision tree) | 2 (gradient-based one side sampling) |
| Maximal depth | 6 | 1 | 15 |
| Learning rate | log2(0.3) | -10 | 0 |
| Early stopping rounds | 7 | 7 | 30 |
| Number of leaves | 15 | 4 | 60 |
| Minimal size of a leave | 10 | 1 | 60 |

**Table 14: Hyperparameters for LGBM.**

The range for the tuning parameters have been previously suggested [62], [63], [64], [65]. Cross-validation was also used to decide whether or not encoding, rebalancing and/or calibration was applied. Encoding was only evaluated in high cardinality variables and then applied as target encoding. Rebalancing, if chosen, was realized through a sequential decision tree generative model [66], [67]. The calibration method, if chosen, was beta calibration [68], [69]. The models were constructed using the sdgm R package [70].

As an NN approach for TSTR, we trained a multi-layer perceptron (MLP). It was built as a sequential classification model with the following architecture using the tensorflow and keras R package [71]:

- an input layer with 16 neurons, using Rectified Linear Unit (RLU) activation,
- a dropout layer with a dropout rate of 30% to prevent overfitting,
- a second hidden layer with 16 neurons, again using RLU activation, and
- an output layer with 1 neuron and a sigmoid activation function for binary classification.

The model was configured to use the Adam optimizer, with binary cross-entropy as the loss function and accuracy as evaluation metric. Training was performed over 50 epochs. A validation split of 20% was used to monitor the model's performance on unseen synthetic data during training. To prevent overfitting, training was stopped as soon as the loss remained stable over two consecutive epochs. We did not further tune hyperparameters as the (untuned) MLP set-up was already comparable to LGBM.

## Replicability of Inferences: Combining Rules

To account for the introduced variance, we combined the estimates of the 10 synthetic datasets using the following combining rule:

Let $q_{i}$be the estimate of the parameter of interest with a variance $v_{i}$ in the synthetic dataset $i$where $i=1...10$. Then, the combined model parameter $\bar{q}_{m}$ was calculated as the mean across the 10 synthetic datasets:

| $\bar{q}_{m}=\frac{1}{10}\sum_{i=1}^{10} q_{i}$ |  |
| --- | --- |

The mean variance across the 10 synthetic datasets is denoted as $\bar{v}_{m}$

| $\bar{v}_{m}=\frac{1}{10}\sum_{i=1}^{10} v_{i}$ |  |
| --- | --- |

And was adjusted as follows:

| $T_{f}=\bar{v}_{m}\left(\frac{n^{R}}{n^{S}}+\frac{1}{10}\right)=\bar{v}_{m}\left(1+\frac{1}{10}\right)$ |  |
| --- | --- |

where $n^{R}$, the size of the real dataset, was equal to $n^{S}$, the size of the synthetic dataset, and the adjusted 95% CI of the parameter could then be computed as $\bar{q}_{m}\pm1.96\sqrt{T_{f}}$.

## Membership Disclosure Vulnerability

Membership disclosure vulnerability was calculated by a partitioning approach [72]. The general way of measuring membership disclosure can be described as follows (see **Figure 1**): A data controller splits the original dataset (i.e., *A_0_*) into a training (i.e., *A_1_*) and holdout (i.e., *A_2_*) dataset. An attack dataset *B_1_* is then built by randomly drawing attack records from *A_1_* (i.e., members) and *A_2_* (i.e., non-members). This mimics an adversary who draws targets from the same population the training data is sampled from. The proportion of records in the attack dataset that are members (i.e., member prevalence) is denoted as *p* and must be determined as shown in [72] to accurately estimate the vulnerability. The distance between each target and its closest synthetic record is calculated. If this distance falls below a certain threshold, it is considered as a match, and the adversary would guess that the target was a member. If the target was indeed a member, the guess is considered as true positive (TP), if it was a non-member as false positive (FP). The members that were guessed as non-members are false negative (FN). Since true negative (TN) guesses, so correct guesses of non-members, are not relevant from an adversary’s perspective, the *F1* score is typically calculated, and reported as relative metric against the *F1* score which results from an adversary randomly guessing membership for their attack dataset.


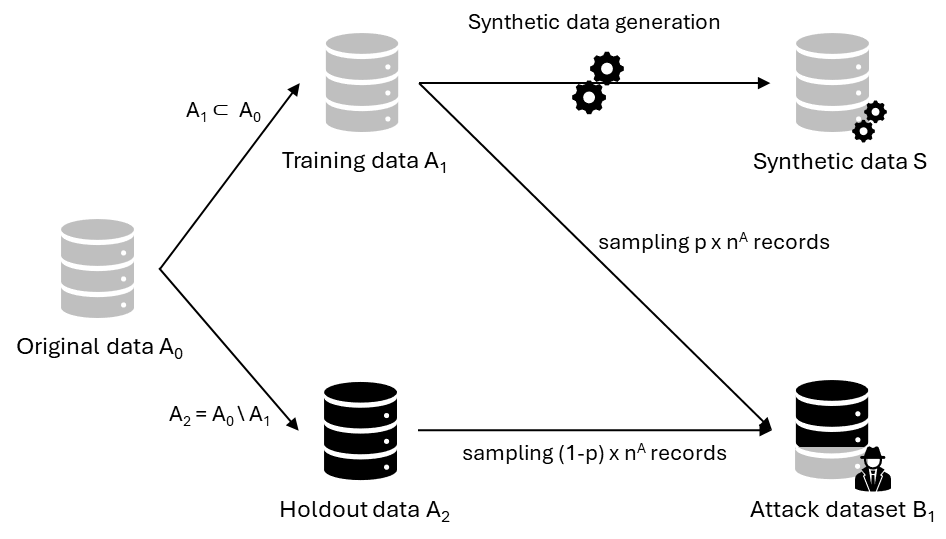


**Figure 2. Partitioning Method for Membership Disclosure.** *A_0_* is split into SDG training data *A_1_* and holdout data *A_2_*. From *A_1_*, *p x n^A^* records are sampled into the attack dataset *B_1_* where *n^A^* is the size of *B_1_*. From *A_2_*, *(1-p) x n^A^* records are sampled into *B_1_*. Adversarial membership guesses are then obtained by matching target records from the attack dataset against synthetic records.

The distance was calculated considering quasi-identifiers (QIs) only. These represent the background knowledge of an adversary [73], [74], [75, p. 29], [76], [77], [78], [79], [80], [81]. We determined QIs for each of the real-world datasets *a priori* in line with published guidelines [73], [82].

| **Medical dataset** | **QIs** |
| --- | --- |
| BORN | birth weight, maternal age, maternal BMI, resident of Ontario, birth month, birth year, public health unit |
| California | age, gender, Hispanic origin, state of residency, race |
| CCHS | gender, age, martial status, province of residency, height, weight, immigrant status, country of birth, ethnicity, BMI |
| COVID-19 | date of reported COVID-19 case, health region, age, gender, residency latitude/longitude, province of residency |
| FAERS | date of event, gender, age, weight |
| Florida | age, gender, state of residency, county of residency, race, zip code |
| MIMIC-III | ethnicity, age, death |
| New York | age, Hispanic origin, state of hospital, state of residency, county of residency, race, zip code |
| NEXOID | country, gender, age, height, weight, BMI, race, immigration status |
| Texas | state of residency, country of residency, gender, race, ethnicity, age |
| Washington 2007 | age, state of residency, county of residency, zip code |
| Washington 2008 | age, gender, Hispanic origin, state of hospital, state of residency, county of residency, race, zip code |

**Table 15. Quasi-identifiers for the Medical Dataset.**

The distribution of the number of QIs across the variants of a medical dataset is presented in Table 16 and the number of QIs against the number of *adjunct* variables is illustrated in Figure 3. Note that an increase in the number of *adjunct* variables did not imply a proportional increase in QIs.

| **Medical dataset** | **Number of QIs Across Population Variants** | | |
| --- | --- | --- | --- |
|  | **Median** | **Q1** | **Q3** |
| BORN | 5 | 4 | 6 |
| California | 3 | 3 | 4 |
| CCHS | 8 | 6 | 10 |
| COVID-19 | 6 | 5 | 7 |
| FAERS | 4 | 4 | 4 |
| Florida | 4 | 4 | 5 |
| MIMIC-III | 2.5 | 2 | 3 |
| New York | 4 | 3 | 5 |
| NEXOID | 6 | 5 | 7 |
| Texas | 6 | 5 | 6 |
| Washington 2007 | 2 | 2 | 3 |
| Washington 2008 | 5 | 4 | 6 |

**Table 16. Distribution of the Number of Quasi-identifiers Across Population Variants.** Q1: first quartile (25^th^ percentile); Q3: third quartile (75^th^ percentile); QIs: Quasi-Identifiers.


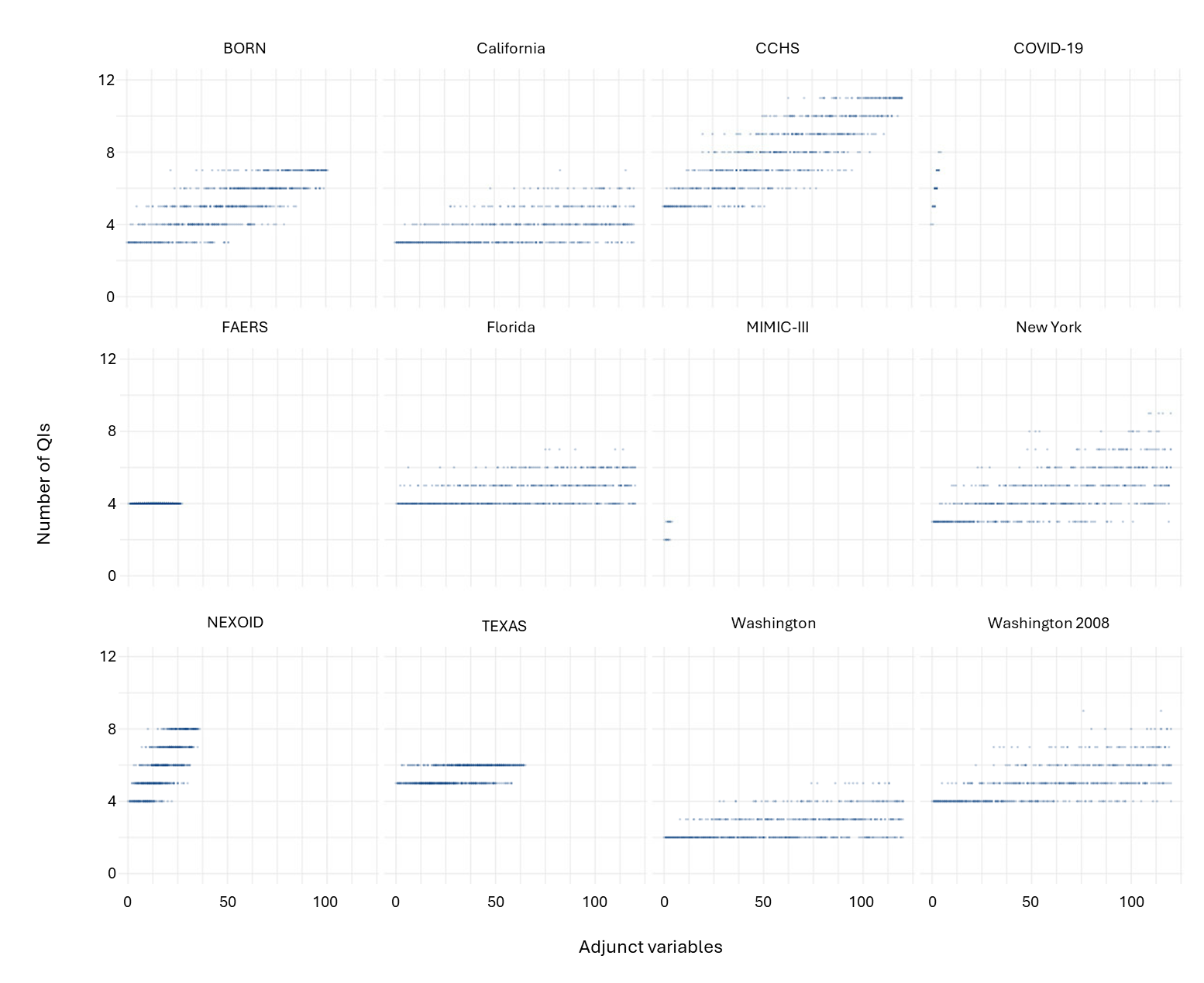


**Figure 3. The Number of Quasi-identifiers When Increasing the Number of Adjunct Variables.** QIs: Quasi-Identifiers.

We also calculated membership disclosure vulnerability based on the entire record (all variables). This is not a very realistic scenario for an adversary, and more importantly, it can deteriorate the matching strategy when matching against synthetic records since mismatches will be more likely the more variables to match [83]. This means entire records is not a worst-case scenario and can be misleading in synthetic data, it can, however, help to detect overfitting.

We implemented distance measurements via Hamming distance and discretized numerical data as described previously in the context of the fidelity metric. The threshold for Hamming distance was set to 0. While, in literature, thresholds for Hamming-based metrics have been set to 2, 3 or 5 [84], this seems not the most realistic strategy for an adversary with QIs as background knowledge. In most QIs, an adversary would very likely have zero tolerance as gender, ethnicity or country are fundamental to the identity. For example, a synthetic record that is male would very likely not be considered as a match with a target who is female. In contrast, a synthetic record that is 25 years old would probably still be considered as a match even if the target is 23 years old. The strategy implemented in this study accounts for these nuances by allowing for some tolerance when matching numerical values through discretizing while being strict about the categorical ones. This is a realistic scenario for an adversary who is confronted with synthetic data.

# DETAILED SYNTHETIC DATA EVALUATION RESULTS

The main manuscript provides effect estimates when modeling the impact of the number of *adjunct* variables on various metrics. In the following, we provide details on the actual measurements of these metrics across the different SDG models and across all variants per medical dataset. More precisely, we report the median and interquartile range (IQR) over all variants of a medical dataset. Given that the number of variables did not relevantly impact evaluation results, this aggregation approach provides a good summary of the evaluation results.

## Fidelity

Across all medical datasets, the ST consistently created high-fidelity synthetic datasets. In other SDG models, fidelity varied depending on the dataset (see Table 17). Variants of the medical dataset FAERS generally presented with lowest fidelity, particularly for RTVAE (median 0.490, IQR [0.470, 0.503]), NFlow (median 0.554, IQR [0.520, 0.587]) and BN (median 0.613, IQR [0.609, 0.617]). MIMIC-III and Washington generated by RTVAE had similar low fidelity (median 0.520, IQR [0.510, 0.527] and median 0.580, IQR [0.558, 0.605], respectively).

| **Model** | **Dataset** | **cluster metric** | | |
| --- | --- | --- | --- | --- |
|  |  | **Median** | **Q1** | **Q3** |
| ST | BORN | 0.998 | 0.998 | 0.999 |
|  | California | 0.984 | 0.978 | 0.987 |
|  | CCHS | 0.986 | 0.981 | 0.989 |
|  | COVID-19 | 0.997 | 0.994 | 0.998 |
|  | FAERS | 0.957 | 0.949 | 0.966 |
|  | Florida | 0.995 | 0.994 | 0.996 |
|  | MIMIC-III | 0.987 | 0.984 | 0.989 |
|  | New York | 0.996 | 0.995 | 0.997 |
|  | NEXOID | 0.991 | 0.973 | 0.996 |
|  | Texas | 0.998 | 0.997 | 0.998 |
|  | Washington | 0.998 | 0.997 | 0.998 |
|  | Washington 2008 | 0.993 | 0.959 | 0.996 |
| BN | BORN | 0.999 | 0.995 | 0.999 |
|  | California | 0.950 | 0.944 | 0.953 |
|  | CCHS | 0.997 | 0.990 | 0.998 |
|  | COVID-19 | 0.884 | 0.882 | 0.890 |
|  | FAERS | 0.613 | 0.609 | 0.617 |
|  | Florida | 0.988 | 0.982 | 0.991 |
|  | MIMIC-III | 0.938 | 0.936 | 0.938 |
|  | New York | 0.979 | 0.973 | 0.986 |
|  | NEXOID | 0.948 | 0.941 | 0.963 |
|  | Texas | 0.992 | 0.991 | 0.994 |
|  | Washington | 0.992 | 0.987 | 0.993 |
|  | Washington 2008 | 0.966 | 0.961 | 0.970 |
| ARF | BORN | 0.991 | 0.989 | 0.993 |
|  | California | 0.938 | 0.890 | 0.975 |
|  | CCHS | 0.987 | 0.984 | 0.990 |
|  | COVID-19 | 0.995 | 0.995 | 0.996 |
|  | FAERS | 0.748 | 0.735 | 0.760 |
|  | Florida | 0.957 | 0.910 | 0.987 |
|  | MIMIC-III | 0.993 | 0.993 | 0.993 |
|  | New York | 0.949 | 0.912 | 0.981 |
|  | NEXOID | 0.981 | 0.980 | 0.983 |
|  | Texas | 0.954 | 0.909 | 0.983 |
|  | Washington | 0.972 | 0.942 | 0.986 |
|  | Washington 2008 | 0.966 | 0.929 | 0.987 |
| CTGAN | BORN | 0.963 | 0.939 | 0.975 |
|  | California | 0.928 | 0.892 | 0.953 |
|  | CCHS | 0.966 | 0.953 | 0.974 |
|  | COVID-19 | 0.829 | 0.729 | 0.865 |
|  | FAERS | 0.702 | 0.667 | 0.727 |
|  | Florida | 0.970 | 0.956 | 0.978 |
|  | MIMIC-III | 0.823 | 0.776 | 0.876 |
|  | New York | 0.954 | 0.926 | 0.971 |
|  | NEXOID | 0.960 | 0.940 | 0.974 |
|  | Texas | 0.975 | 0.967 | 0.981 |
|  | Washington | 0.966 | 0.950 | 0.975 |
|  | Washington 2008 | 0.944 | 0.892 | 0.969 |
| TVAE | BORN | 0.940 | 0.932 | 0.951 |
|  | California | 0.937 | 0.919 | 0.952 |
|  | CCHS | 0.972 | 0.969 | 0.975 |
|  | COVID-19 | 0.852 | 0.781 | 0.949 |
|  | FAERS | 0.737 | 0.719 | 0.749 |
|  | Florida | 0.974 | 0.971 | 0.978 |
|  | MIMIC-III | 0.925 | 0.868 | 0.951 |
|  | New York | 0.959 | 0.946 | 0.967 |
|  | NEXOID | 0.965 | 0.951 | 0.974 |
|  | Texas | 0.977 | 0.974 | 0.980 |
|  | Washington | 0.967 | 0.962 | 0.972 |
|  | Washington 2008 | 0.961 | 0.945 | 0.969 |
| RTVAE | BORN | 0.941 | 0.932 | 0.951 |
|  | California | 0.677 | 0.662 | 0.691 |
|  | CCHS | 0.959 | 0.952 | 0.964 |
|  | COVID-19 | 0.718 | 0.647 | 0.891 |
|  | FAERS | 0.490 | 0.470 | 0.503 |
|  | Florida | 0.781 | 0.764 | 0.797 |
|  | MIMIC-III | 0.520 | 0.510 | 0.527 |
|  | New York | 0.726 | 0.716 | 0.737 |
|  | NEXOID | 0.936 | 0.905 | 0.967 |
|  | Texas | 0.929 | 0.922 | 0.936 |
|  | Washington | 0.580 | 0.558 | 0.605 |
|  | Washington 2008 | 0.709 | 0.696 | 0.722 |
| NFlow | BORN | 0.854 | 0.816 | 0.888 |
|  | California | 0.870 | 0.840 | 0.893 |
|  | CCHS | 0.884 | 0.854 | 0.907 |
|  | COVID-19 | 0.771 | 0.704 | 0.829 |
|  | FAERS | 0.554 | 0.520 | 0.587 |
|  | Florida | 0.905 | 0.876 | 0.929 |
|  | MIMIC-III | 0.824 | 0.782 | 0.842 |
|  | New York | 0.889 | 0.858 | 0.913 |
|  | NEXOID | 0.877 | 0.844 | 0.907 |
|  | Texas | 0.890 | 0.864 | 0.916 |
|  | Washington | 0.928 | 0.902 | 0.949 |
|  | Washington 2008 | 0.891 | 0.863 | 0.913 |

**Table 17.** **Results for *Core* Fidelity Across Variants per SDG Model and Medical Dataset.** Fidelity was measured by cluster analysis as described in the methods. The range of this metric was scaled between 0-1 with 1 being maximum fidelity. The distribution across variants per medical dataset is reported as median, 1^st^ (Q1) and 3^rd^ quartile (Q3).

## Downstream Utility

For downstream utility, the performance was dependent on the SDG model and the medical dataset but also whether it was utility in AI/ML predictive tasks or replicability of inferences.

For AI/ML predictive tasks, MLP generally achieved better discriminative performance than LGBM. As with fidelity, performance differed between SDG models and medical datasets. In ST, the differences between the median TSTR and TRTR were lower than 0.1 across all medical datasets whether it was LGBM or MLP. Differences between the median TSTR and TRTR were similarly low for both AI/ML predictive models in synthetic data generated by ARF. For other SDG models, the AUROC difference between median TSTR and TRTR exceeded 0.1 in several instances: BN had a maximum difference of 0.41 for COVID-19 in LGBM, CTGAN a maximum difference of 0.304 for COVID-19 in LGBM, TVAE a maximum difference of 0.236 for COVID-19 in LGBM, RTVAE a maximum difference of 0.199 in COVID-19 in LGBM and NFlow a maximum of 0.267 in New York for LGBM. Details are depicted in Table 18. Notably, the median TSTR in MLP outperformed TRTR for the following SDG model and dataset combinations: BN and CCHS (-0.003), ARF and NEXOID (-0.01), CTGAN and MIMIC-III (-0.001), TVAE and NEXOID (-0.006), and RTVAE and NEXOID (-0.003).

For replicability of inferences, evaluation results were mixed. While, for example, the proportion of estimate agreement across variants of MIMIC-III generated by ST was low (18.8%), the corresponding decision agreement, standardized difference and 95% CI overlap were notably higher (i.e., 62.5% variants with decision agreement, 100% with positive standardized difference and median 95% CI overlap of 76.2%). The following SDG model and dataset combinations achieved estimate agreement in less than 10% of the population variants: BN and Florida (4.2%), BN and MIMIC-III (6.3%), BN and New York (0.2%), ARF and Florida (4.5%), ARF and MIMIC-III (0%), ARF and New York (8.2%), ARF and NEXOID (2.7%), ARF and Texas
(0%), ARF and Washington (0%), CTGAN and Florida (7%), CTGAN and New York (4.5%), CTGAN and Texas (0.2%), CTGAN and Washington (3%), TVAE and Florida (6.3%), TVAE and New York (0.3%), TVAE and Texas (6.5%), TVAE and Washington (0.8%), RTVAE and MIMIC-III (0%), and NFlow and Texas (8.4%). Similarly, decision agreement, standardized difference and 95% CI overlap have mixed results (see details in Table 19).

These results suggest that high fidelity or high discriminative performance in AI/ML prediction does not necessarily translate into reproducibility of inferences.

| **Model** | **Dataset** | **LGBM TSTR** | | | | **MLP TSTR** | | | |
| --- | --- | --- | --- | --- | --- | --- | --- | --- | --- |
|  |  | **Median** | **Q1** | **Q3** | **TRTR** | **Median** | **Q1** | **Q3** | **TRTR** |
| ST | BORN | 0.918 | 0.917 | 0.919 | 0.923 | 0.882 | 0.875 | 0.887 | 0.896 |
|  | California | 0.760 | 0.756 | 0.763 | 0.810 | 0.846 | 0.844 | 0.848 | 0.854 |
|  | CCHS | 0.611 | 0.603 | 0.618 | 0.708 | 0.697 | 0.695 | 0.699 | 0.698 |
|  | COVID-19 | 0.936 | 0.933 | 0.940 | 0.957 | 0.884 | 0.872 | 0.901 | 0.931 |
|  | FAERS | 0.587 | 0.578 | 0.594 | 0.663 | 0.912 | 0.900 | 0.922 | 0.928 |
|  | Florida | 0.692 | 0.688 | 0.697 | 0.750 | 0.835 | 0.833 | 0.837 | 0.837 |
|  | MIMIC-III | 0.582 | 0.573 | 0.589 | 0.654 | 0.534 | 0.530 | 0.538 | 0.534 |
|  | New York | 0.740 | 0.732 | 0.745 | 0.806 | 0.863 | 0.861 | 0.866 | 0.859 |
|  | NEXOID | 0.709 | 0.704 | 0.714 | 0.730 | 0.702 | 0.694 | 0.707 | 0.681 |
|  | Texas | 0.788 | 0.784 | 0.790 | 0.810 | 0.811 | 0.810 | 0.812 | 0.813 |
|  | Washington | 0.735 | 0.732 | 0.738 | 0.784 | 0.862 | 0.860 | 0.864 | 0.870 |
|  | Washington 2008 | 0.743 | 0.735 | 0.750 | 0.808 | 0.870 | 0.868 | 0.871 | 0.877 |
| BN | BORN | 0.904 | 0.902 | 0.909 | 0.923 | 0.886 | 0.858 | 0.889 | 0.896 |
|  | California | 0.697 | 0.693 | 0.705 | 0.810 | 0.839 | 0.835 | 0.841 | 0.854 |
|  | CCHS | 0.660 | 0.656 | 0.664 | 0.708 | 0.701 | 0.689 | 0.702 | 0.698 |
|  | COVID-19 | 0.547 | 0.527 | 0.566 | 0.957 | 0.773 | 0.747 | 0.798 | 0.931 |
|  | FAERS | 0.556 | 0.551 | 0.562 | 0.663 | 0.822 | 0.796 | 0.843 | 0.928 |
|  | Florida | 0.626 | 0.620 | 0.636 | 0.750 | 0.822 | 0.814 | 0.828 | 0.837 |
|  | MIMIC-III | 0.562 | 0.561 | 0.567 | 0.654 | 0.526 | 0.522 | 0.530 | 0.534 |
|  | New York | 0.665 | 0.657 | 0.677 | 0.806 | 0.849 | 0.845 | 0.852 | 0.859 |
|  | NEXOID | 0.678 | 0.667 | 0.686 | 0.730 | 0.673 | 0.657 | 0.684 | 0.681 |
|  | Texas | 0.762 | 0.759 | 0.764 | 0.810 | 0.798 | 0.796 | 0.801 | 0.813 |
|  | Washington | 0.658 | 0.653 | 0.663 | 0.784 | 0.849 | 0.844 | 0.854 | 0.870 |
|  | Washington 2008 | 0.690 | 0.686 | 0.710 | 0.808 | 0.859 | 0.856 | 0.861 | 0.877 |
| ARF | BORN | 0.910 | 0.907 | 0.912 | 0.923 | 0.865 | 0.859 | 0.870 | 0.896 |
|  | California | 0.728 | 0.721 | 0.736 | 0.810 | 0.835 | 0.828 | 0.839 | 0.854 |
|  | CCHS | 0.685 | 0.682 | 0.689 | 0.708 | 0.690 | 0.687 | 0.693 | 0.698 |
|  | COVID-19 | 0.931 | 0.917 | 0.936 | 0.957 | 0.831 | 0.826 | 0.853 | 0.931 |
|  | FAERS | 0.578 | 0.571 | 0.584 | 0.663 | 0.818 | 0.790 | 0.843 | 0.928 |
|  | Florida | 0.650 | 0.643 | 0.657 | 0.750 | 0.795 | 0.786 | 0.803 | 0.837 |
|  | MIMIC-III | 0.577 | 0.574 | 0.583 | 0.654 | 0.523 | 0.519 | 0.526 | 0.534 |
|  | New York | 0.692 | 0.680 | 0.704 | 0.806 | 0.821 | 0.811 | 0.829 | 0.859 |
|  | NEXOID | 0.705 | 0.702 | 0.709 | 0.730 | 0.691 | 0.686 | 0.696 | 0.681 |
|  | Texas | 0.759 | 0.757 | 0.761 | 0.810 | 0.798 | 0.791 | 0.804 | 0.813 |
|  | Washington | 0.684 | 0.679 | 0.688 | 0.784 | 0.834 | 0.826 | 0.841 | 0.870 |
|  | Washington 2008 | 0.709 | 0.705 | 0.714 | 0.808 | 0.845 | 0.840 | 0.851 | 0.877 |
| CTGAN | BORN | 0.899 | 0.892 | 0.904 | 0.923 | 0.855 | 0.846 | 0.862 | 0.896 |
|  | California | 0.646 | 0.618 | 0.658 | 0.810 | 0.819 | 0.810 | 0.826 | 0.854 |
|  | CCHS | 0.681 | 0.675 | 0.685 | 0.708 | 0.692 | 0.689 | 0.695 | 0.698 |
|  | COVID-19 | 0.653 | 0.610 | 0.674 | 0.957 | 0.657 | 0.621 | 0.664 | 0.931 |
|  | FAERS | 0.536 | 0.524 | 0.546 | 0.663 | 0.789 | 0.761 | 0.816 | 0.928 |
|  | Florida | 0.613 | 0.580 | 0.626 | 0.750 | 0.816 | 0.808 | 0.823 | 0.837 |
|  | MIMIC-III | 0.558 | 0.551 | 0.565 | 0.654 | 0.535 | 0.527 | 0.542 | 0.534 |
|  | New York | 0.633 | 0.606 | 0.646 | 0.806 | 0.836 | 0.829 | 0.843 | 0.859 |
|  | NEXOID | 0.666 | 0.659 | 0.673 | 0.730 | 0.675 | 0.669 | 0.681 | 0.681 |
|  | Texas | 0.733 | 0.719 | 0.741 | 0.810 | 0.786 | 0.781 | 0.791 | 0.813 |
|  | Washington | 0.645 | 0.638 | 0.651 | 0.784 | 0.846 | 0.840 | 0.850 | 0.870 |
|  | Washington 2008 | 0.661 | 0.646 | 0.671 | 0.808 | 0.842 | 0.832 | 0.849 | 0.877 |
| TVAE | BORN | 0.908 | 0.903 | 0.911 | 0.923 | 0.866 | 0.857 | 0.872 | 0.896 |
|  | California | 0.660 | 0.649 | 0.670 | 0.810 | 0.815 | 0.808 | 0.821 | 0.854 |
|  | CCHS | 0.684 | 0.680 | 0.687 | 0.708 | 0.696 | 0.694 | 0.698 | 0.698 |
|  | COVID-19 | 0.721 | 0.601 | 0.785 | 0.957 | 0.706 | 0.652 | 0.727 | 0.931 |
|  | FAERS | 0.564 | 0.556 | 0.573 | 0.663 | 0.841 | 0.807 | 0.871 | 0.928 |
|  | Florida | 0.619 | 0.605 | 0.627 | 0.750 | 0.814 | 0.807 | 0.820 | 0.837 |
|  | MIMIC-III | 0.556 | 0.549 | 0.562 | 0.654 | 0.527 | 0.523 | 0.531 | 0.534 |
|  | New York | 0.644 | 0.634 | 0.653 | 0.806 | 0.829 | 0.822 | 0.836 | 0.859 |
|  | NEXOID | 0.676 | 0.670 | 0.680 | 0.730 | 0.687 | 0.681 | 0.692 | 0.681 |
|  | Texas | 0.749 | 0.745 | 0.752 | 0.810 | 0.779 | 0.775 | 0.783 | 0.813 |
|  | Washington | 0.649 | 0.641 | 0.654 | 0.784 | 0.844 | 0.839 | 0.849 | 0.870 |
|  | Washington 2008 | 0.686 | 0.680 | 0.690 | 0.808 | 0.835 | 0.827 | 0.841 | 0.877 |
| RTVAE | BORN | 0.907 | 0.903 | 0.910 | 0.923 | 0.866 | 0.857 | 0.873 | 0.896 |
|  | California | 0.642 | 0.623 | 0.655 | 0.810 | 0.712 | 0.696 | 0.730 | 0.854 |
|  | CCHS | 0.659 | 0.653 | 0.664 | 0.708 | 0.696 | 0.693 | 0.699 | 0.698 |
|  | COVID-19 | 0.758 | 0.673 | 0.810 | 0.957 | 0.664 | 0.630 | 0.715 | 0.931 |
|  | FAERS | 0.544 | 0.527 | 0.555 | 0.663 | 0.683 | 0.652 | 0.721 | 0.928 |
|  | Florida | 0.607 | 0.596 | 0.616 | 0.750 | 0.737 | 0.724 | 0.753 | 0.837 |
|  | MIMIC-III | 0.517 | 0.502 | 0.522 | 0.654 | 0.526 | 0.521 | 0.532 | 0.534 |
|  | New York | 0.641 | 0.629 | 0.649 | 0.806 | 0.722 | 0.705 | 0.741 | 0.859 |
|  | NEXOID | 0.665 | 0.646 | 0.673 | 0.730 | 0.684 | 0.674 | 0.689 | 0.681 |
|  | Texas | 0.721 | 0.712 | 0.726 | 0.810 | 0.775 | 0.770 | 0.780 | 0.813 |
|  | Washington | 0.611 | 0.599 | 0.617 | 0.784 | 0.790 | 0.774 | 0.806 | 0.870 |
|  | Washington 2008 | 0.650 | 0.638 | 0.658 | 0.808 | 0.776 | 0.763 | 0.789 | 0.877 |
| NFlow | BORN | 0.839 | 0.791 | 0.869 | 0.923 | 0.801 | 0.754 | 0.830 | 0.896 |
|  | California | 0.586 | 0.562 | 0.610 | 0.810 | 0.806 | 0.792 | 0.818 | 0.854 |
|  | CCHS | 0.623 | 0.577 | 0.642 | 0.708 | 0.674 | 0.667 | 0.679 | 0.698 |
|  | COVID-19 | 0.821 | 0.727 | 0.843 | 0.957 | 0.705 | 0.636 | 0.758 | 0.931 |
|  | FAERS | 0.523 | 0.506 | 0.539 | 0.663 | 0.812 | 0.776 | 0.853 | 0.928 |
|  | Florida | 0.537 | 0.522 | 0.557 | 0.750 | 0.797 | 0.779 | 0.811 | 0.837 |
|  | MIMIC-III | 0.552 | 0.549 | 0.558 | 0.654 | 0.522 | 0.518 | 0.526 | 0.534 |
|  | New York | 0.539 | 0.525 | 0.561 | 0.806 | 0.816 | 0.794 | 0.833 | 0.859 |
|  | NEXOID | 0.652 | 0.630 | 0.668 | 0.730 | 0.660 | 0.646 | 0.676 | 0.681 |
|  | Texas | 0.691 | 0.672 | 0.705 | 0.810 | 0.778 | 0.772 | 0.784 | 0.813 |
|  | Washington | 0.575 | 0.556 | 0.593 | 0.784 | 0.842 | 0.828 | 0.850 | 0.870 |
|  | Washington 2008 | 0.597 | 0.577 | 0.620 | 0.808 | 0.836 | 0.824 | 0.844 | 0.877 |

**Table 18.** **Results for TSTR as Downstream Utility Across Variants per SDG Model and Medical Dataset.** Downstream utility was assessed as AUROC via TSTR using LGBM and MLP for prognostic modeling. The distribution across variants per medical dataset is reported as median, 1^st^ (Q1) and 3^rd^ quartile (Q3). The AUROC derived from the real training data is also indicated (i.e., TRTR).

| **Model** | **Dataset** | **Estimate Agreement** | | **Decision Agreement** | | **Standardized Difference** | | **95% CI overlap** | | |
| --- | --- | --- | --- | --- | --- | --- | --- | --- | --- | --- |
|  |  | **Count** | **%** | **Count** | **%** | **Count** | **%** | **Median** | **Q1** | **Q3** |
| ST | BORN | 473 | 67.6 | 173 | 24.7 | 347 | 49.6 | 0.290 | 0.149 | 0.482 |
|  | California | 597 | 99.3 | 601 | 100.0 | 601 | 100.0 | 0.898 | 0.831 | 0.949 |
|  | CCHS | 631 | 87.3 | 569 | 78.7 | 622 | 86.0 | 0.795 | 0.565 | 0.907 |
|  | COVID-19 | 31 | 96.9 | 32 | 100.0 | 32 | 100.0 | 0.851 | 0.808 | 0.907 |
|  | FAERS | 613 | 99.8 | 558 | 90.9 | 613 | 99.8 | 0.667 | 0.585 | 0.740 |
|  | Florida | 593 | 98.7 | 327 | 54.4 | 540 | 89.9 | 0.538 | 0.412 | 0.664 |
|  | MIMIC-III | 3 | 18.8 | 10 | 62.5 | 16 | 100.0 | 0.762 | 0.462 | 0.873 |
|  | New York | 281 | 46.8 | 540 | 89.9 | 598 | 99.5 | 0.711 | 0.611 | 0.808 |
|  | NEXOID | 296 | 47.6 | 380 | 61.1 | 544 | 87.5 | 0.556 | 0.406 | 0.739 |
|  | Texas | 187 | 29.1 | 601 | 93.6 | 622 | 96.9 | 0.501 | 0.501 | 0.598 |
|  | Washington | 582 | 96.8 | 547 | 91.0 | 586 | 97.5 | 0.781 | 0.645 | 0.896 |
|  | Washington 2008 | 601 | 100.0 | 601 | 100.0 | 601 | 100.0 | 0.930 | 0.884 | 0.965 |
| BN | BORN | 489 | 69.9 | 483 | 69.0 | 483 | 69.0 | 0.907 | 0.000 | 0.959 |
|  | California | 570 | 94.8 | 601 | 100.0 | 601 | 100.0 | 0.812 | 0.747 | 0.880 |
|  | CCHS | 578 | 79.9 | 619 | 85.6 | 706 | 97.6 | 0.780 | 0.648 | 0.864 |
|  | COVID-19 | 32 | 100.0 | 32 | 100.0 | 32 | 100.0 | 0.789 | 0.752 | 0.847 |
|  | FAERS | 595 | 96.9 | 595 | 96.9 | 595 | 96.9 | 0.500 | 0.500 | 0.621 |
|  | Florida | 25 | 4.2 | 229 | 38.1 | 395 | 65.7 | 0.485 | 0.233 | 0.689 |
|  | MIMIC-III | 1 | 6.3 | 16 | 100.0 | 16 | 100.0 | 0.773 | 0.709 | 0.787 |
|  | New York | 1 | 0.2 | 1 | 0.2 | 1 | 0.2 | 0.000 | 0.000 | 0.000 |
|  | NEXOID | 313 | 50.3 | 367 | 59.0 | 562 | 90.4 | 0.579 | 0.386 | 0.740 |
|  | Texas | 318 | 49.5 | 591 | 92.1 | 592 | 92.2 | 0.503 | 0.501 | 0.914 |
|  | Washington | 11 | 1.8 | 234 | 38.9 | 400 | 66.6 | 0.464 | 0.045 | 0.576 |
|  | Washington 2008 | 601 | 100.0 | 601 | 100.0 | 601 | 100.0 | 0.911 | 0.855 | 0.952 |
| ARF | BORN | 507 | 72.4 | 0 | 0.0 | 27 | 3.9 | 0.080 | 0.015 | 0.155 |
|  | California | 599 | 99.7 | 601 | 100.0 | 601 | 100.0 | 0.878 | 0.838 | 0.924 |
|  | CCHS | 376 | 52.0 | 696 | 96.3 | 722 | 99.9 | 0.672 | 0.588 | 0.778 |
|  | COVID-19 | 32 | 100.0 | 24 | 75.0 | 32 | 100.0 | 0.551 | 0.495 | 0.597 |
|  | FAERS | 614 | 100.0 | 497 | 80.9 | 614 | 100.0 | 0.777 | 0.682 | 0.861 |
|  | Florida | 27 | 4.5 | 499 | 83.0 | 594 | 98.8 | 0.729 | 0.618 | 0.852 |
|  | MIMIC-III | 0 | 0.0 | 16 | 100.0 | 16 | 100.0 | 0.710 | 0.680 | 0.761 |
|  | New York | 49 | 8.2 | 218 | 36.3 | 450 | 74.9 | 0.485 | 0.291 | 0.628 |
|  | NEXOID | 17 | 2.7 | 33 | 5.3 | 210 | 33.8 | 0.236 | 0.148 | 0.330 |
|  | Texas | 0 | 0.0 | 609 | 94.9 | 612 | 95.3 | 0.501 | 0.501 | 0.501 |
|  | Washington | 0 | 0.0 | 1 | 0.2 | 9 | 1.5 | 0.000 | 0.000 | 0.000 |
|  | Washington 2008 | 601 | 100.0 | 601 | 100.0 | 601 | 100.0 | 0.887 | 0.843 | 0.933 |
| CTGAN | BORN | 201 | 28.7 | 24 | 3.4 | 87 | 12.4 | 0.000 | 0.000 | 0.154 |
|  | California | 415 | 69.1 | 533 | 88.7 | 589 | 98.0 | 0.788 | 0.632 | 0.882 |
|  | CCHS | 653 | 90.3 | 575 | 79.5 | 651 | 90.0 | 0.742 | 0.533 | 0.871 |
|  | COVID-19 | 32 | 100.0 | 32 | 100.0 | 32 | 100.0 | 0.706 | 0.655 | 0.785 |
|  | FAERS | 608 | 99.0 | 600 | 97.7 | 608 | 99.0 | 0.832 | 0.772 | 0.885 |
|  | Florida | 42 | 7.0 | 185 | 30.8 | 364 | 60.6 | 0.393 | 0.120 | 0.630 |
|  | MIMIC-III | 3 | 18.8 | 14 | 87.5 | 16 | 100.0 | 0.747 | 0.542 | 0.896 |
|  | New York | 27 | 4.5 | 55 | 9.2 | 128 | 21.3 | 0.000 | 0.000 | 0.239 |
|  | NEXOID | 416 | 66.9 | 374 | 60.1 | 550 | 88.4 | 0.584 | 0.414 | 0.732 |
|  | Texas | 1 | 0.2 | 571 | 88.9 | 597 | 93.0 | 0.502 | 0.491 | 0.629 |
|  | Washington | 18 | 3.0 | 41 | 6.8 | 103 | 17.1 | 0.000 | 0.000 | 0.178 |
|  | Washington 2008 | 594 | 98.8 | 600 | 99.8 | 600 | 99.8 | 0.889 | 0.846 | 0.928 |
| TVAE | BORN | 296 | 42.3 | 413 | 59.0 | 525 | 75.0 | 0.478 | 0.290 | 0.636 |
|  | California | 478 | 79.5 | 535 | 89.0 | 591 | 98.3 | 0.777 | 0.631 | 0.874 |
|  | CCHS | 718 | 99.3 | 285 | 39.4 | 405 | 56.0 | 0.362 | 0.019 | 0.699 |
|  | COVID-19 | 32 | 100.0 | 31 | 96.9 | 32 | 100.0 | 0.536 | 0.501 | 0.670 |
|  | FAERS | 595 | 96.9 | 579 | 94.3 | 589 | 95.9 | 0.637 | 0.556 | 0.679 |
|  | Florida | 38 | 6.3 | 307 | 51.1 | 480 | 79.9 | 0.599 | 0.354 | 0.798 |
|  | MIMIC-III | 4 | 25.0 | 10 | 62.5 | 11 | 68.8 | 0.724 | 0.239 | 0.803 |
|  | New York | 2 | 0.3 | 10 | 1.7 | 54 | 9.0 | 0.000 | 0.000 | 0.125 |
|  | NEXOID | 462 | 74.3 | 461 | 74.1 | 587 | 94.4 | 0.690 | 0.501 | 0.843 |
|  | Texas | 42 | 6.5 | 569 | 88.6 | 571 | 88.9 | 0.500 | 0.500 | 0.501 |
|  | Washington | 5 | 0.8 | 22 | 3.7 | 53 | 8.8 | 0.000 | 0.000 | 0.000 |
|  | Washington 2008 | 598 | 99.5 | 599 | 99.7 | 600 | 99.8 | 0.838 | 0.790 | 0.876 |
| RTVAE | BORN | 321 | 45.9 | 409 | 58.4 | 509 | 72.7 | 0.490 | 0.257 | 0.679 |
|  | California | 328 | 54.6 | 425 | 70.7 | 502 | 83.5 | 0.608 | 0.411 | 0.809 |
|  | CCHS | 679 | 93.9 | 363 | 50.2 | 458 | 63.3 | 0.480 | 0.110 | 0.736 |
|  | COVID-19 | 32 | 100.0 | 32 | 100.0 | 32 | 100.0 | 0.501 | 0.501 | 0.501 |
|  | FAERS | 610 | 99.3 | 611 | 99.5 | 611 | 99.5 | 0.505 | 0.505 | 0.505 |
|  | Florida | 517 | 86.0 | 520 | 86.5 | 540 | 89.9 | 0.501 | 0.501 | 0.501 |
|  | MIMIC-III | 0 | 0.0 | 14 | 87.5 | 16 | 100.0 | 0.501 | 0.501 | 0.501 |
|  | New York | 519 | 86.5 | 525 | 87.5 | 533 | 88.8 | 0.501 | 0.501 | 0.501 |
|  | NEXOID | 407 | 65.4 | 451 | 72.5 | 556 | 89.4 | 0.668 | 0.472 | 0.831 |
|  | Texas | 235 | 36.6 | 397 | 61.8 | 397 | 61.8 | 0.500 | 0.000 | 0.501 |
|  | Washington | 522 | 86.9 | 524 | 87.2 | 533 | 88.7 | 0.501 | 0.501 | 0.501 |
|  | Washington 2008 | 596 | 99.2 | 598 | 99.5 | 599 | 99.7 | 0.777 | 0.724 | 0.826 |
| NFlow | BORN | 147 | 21.1 | 40 | 5.7 | 99 | 14.2 | 0.000 | 0.000 | 0.114 |
|  | California | 420 | 69.9 | 544 | 90.5 | 589 | 98.0 | 0.793 | 0.682 | 0.861 |
|  | CCHS | 97 | 13.7 | 229 | 32.3 | 354 | 49.9 | 0.291 | 0.098 | 0.553 |
|  | COVID-19 | 26 | 81.3 | 21 | 65.6 | 28 | 87.5 | 0.678 | 0.480 | 0.755 |
|  | FAERS | 570 | 92.8 | 395 | 64.3 | 572 | 93.2 | 0.565 | 0.500 | 0.729 |
|  | Florida | 157 | 26.2 | 464 | 77.3 | 580 | 96.7 | 0.761 | 0.703 | 0.794 |
|  | MIMIC-III | 5 | 31.3 | 7 | 43.8 | 10 | 62.5 | 0.550 | 0.252 | 0.620 |
|  | New York | 165 | 27.6 | 432 | 72.4 | 580 | 97.2 | 0.745 | 0.676 | 0.772 |
|  | NEXOID | 172 | 27.7 | 257 | 41.3 | 413 | 66.4 | 0.420 | 0.209 | 0.626 |
|  | Texas | 54 | 8.4 | 33 | 5.1 | 43 | 6.7 | 0.000 | 0.000 | 0.000 |
|  | Washington | 219 | 36.6 | 341 | 57.0 | 472 | 78.9 | 0.648 | 0.365 | 0.826 |
|  | Washington 2008 | 554 | 92.5 | 580 | 96.8 | 596 | 99.5 | 0.804 | 0.757 | 0.855 |

**Table 19.** **Results for Replicability of Inferences as Downstream Utility Across Variants per SDG Model and Medical Dataset.** Downstream utility was assessed as replicability with the four metrics described in the main manuscript. Decision agreement, estimate agreement and standardized difference are binary with 1 indicating agreement or, in the case of standardized difference, consistency with the null hypothesis of no difference. 95% CI overlap ranges between 0 and 1 and indicates the proportion of overlap between the synthetic and real 95% CI. The distribution across variants per medical dataset is reported as count and percentile of agreement in the case of the binary metrics, and as median, 1^st^ (Q1) and 3^rd^ quartile (Q3) in the case of 95% CI overlap.

## Membership Disclosure Vulnerability

For membership disclosure vulnerability, we provide details on the distribution of the actual measurements based on QIs (see Table 20). Across all SDG models and medical datasets, the values of membership disclosure vulnerability were low with a maximum median vulnerability of 0.004 across the variants of Washington 2008 generated by ST and across the variants of CCHS generated by BN. Among the variants of CCHS generated by BN was also the highest vulnerability across all SDG models and medical datasets (maximum 0.04).

Membership disclosure vulnerability was additionally calculated based on all variables. This is meant to detect overfitting but also serves as a robustness analysis to better understand the sensitivity of our results to the selection of QIs. Similar to the results based on QIs, higher values could be observed in variants of CCHS generated by BN (maximum 0.27) (see Figure 5). All other variants had residual membership disclosure vulnerabilities below 0.2 (see Figure 4, Figure 6, Figure 7, Figure 8, Figure 9 and Figure 10).

| **Model** | **Dataset** | ***Core + adjunct* dataset** | | | ***Core* dataset** | | |
| --- | --- | --- | --- | --- | --- | --- | --- |
|  |  | **Median** | **Q1** | **Q3** | **Median** | **Q1** | **Q3** |
| ST | BORN | 0.000 | 0.000 | 0.001 | 0.000 | 0.000 | 0.000 |
|  | California | 0.000 | 0.000 | 0.000 | 0.000 | 0.000 | 0.000 |
|  | CCHS | -0.018 | -0.021 | -0.014 | -0.012 | -0.012 | -0.012 |
|  | COVID-19 | 0.000 | 0.000 | 0.001 | 0.000 | 0.000 | 0.000 |
|  | FAERS | 0.001 | 0.001 | 0.001 | 0.001 | 0.001 | 0.001 |
|  | Florida | 0.003 | 0.003 | 0.003 | 0.003 | 0.003 | 0.003 |
|  | MIMIC-III | -0.003 | -0.003 | -0.001 | -0.001 | -0.001 | -0.001 |
|  | New York | 0.002 | 0.002 | 0.002 | 0.002 | 0.002 | 0.002 |
|  | NEXOID | 0.000 | 0.000 | 0.001 | 0.000 | 0.000 | 0.000 |
|  | Texas | 0.000 | 0.000 | 0.000 | 0.000 | 0.000 | 0.000 |
|  | Washington | 0.002 | 0.001 | 0.002 | 0.002 | 0.001 | 0.002 |
|  | Washington 2008 | 0.004 | 0.004 | 0.005 | 0.004 | 0.004 | 0.005 |
| BN | BORN | 0.001 | 0.000 | 0.003 | 0.000 | 0.000 | 0.000 |
|  | California | 0.000 | 0.000 | 0.000 | 0.000 | 0.000 | 0.000 |
|  | CCHS | 0.004 | 0.001 | 0.024 | 0.000 | 0.000 | 0.000 |
|  | COVID-19 | 0.000 | 0.000 | 0.000 | 0.000 | 0.000 | 0.000 |
|  | FAERS | 0.000 | 0.000 | 0.000 | 0.000 | 0.000 | 0.000 |
|  | Florida | 0.001 | 0.000 | 0.001 | 0.001 | 0.000 | 0.001 |
|  | MIMIC-III | -0.004 | -0.004 | -0.003 | -0.003 | -0.003 | -0.003 |
|  | New York | 0.001 | 0.001 | 0.001 | 0.001 | 0.001 | 0.001 |
|  | NEXOID | 0.000 | 0.000 | 0.001 | 0.000 | 0.000 | 0.000 |
|  | Texas | 0.001 | 0.001 | 0.001 | 0.001 | 0.000 | 0.001 |
|  | Washington | 0.000 | 0.000 | 0.001 | 0.000 | 0.000 | 0.001 |
|  | Washington 2008 | 0.000 | 0.000 | 0.002 | 0.000 | 0.000 | 0.002 |
| ARF | BORN | 0.000 | 0.000 | 0.000 | 0.000 | 0.000 | 0.000 |
|  | California | 0.000 | 0.000 | 0.000 | 0.000 | 0.000 | 0.000 |
|  | CCHS | -0.001 | -0.007 | 0.000 | 0.000 | 0.000 | 0.000 |
|  | COVID-19 | 0.000 | 0.000 | 0.000 | 0.000 | 0.000 | 0.000 |
|  | FAERS | 0.001 | 0.001 | 0.001 | 0.001 | 0.001 | 0.001 |
|  | Florida | 0.000 | 0.000 | 0.000 | 0.000 | 0.000 | 0.000 |
|  | MIMIC-III | -0.002 | -0.003 | -0.001 | -0.001 | -0.001 | -0.001 |
|  | New York | 0.001 | 0.000 | 0.001 | 0.001 | 0.001 | 0.001 |
|  | NEXOID | 0.000 | 0.000 | 0.000 | 0.000 | 0.000 | 0.000 |
|  | Texas | 0.000 | 0.000 | 0.000 | 0.000 | 0.000 | 0.000 |
|  | Washington | 0.000 | 0.000 | 0.000 | 0.000 | 0.000 | 0.000 |
|  | Washington 2008 | 0.000 | -0.001 | 0.000 | 0.000 | 0.000 | 0.000 |
| CTGAN | BORN | 0.000 | 0.000 | 0.000 | 0.000 | 0.000 | 0.000 |
|  | California | 0.000 | 0.000 | 0.000 | 0.000 | 0.000 | 0.000 |
|  | CCHS | 0.000 | -0.006 | 0.000 | 0.000 | 0.000 | 0.000 |
|  | COVID-19 | 0.000 | -0.001 | 0.000 | 0.000 | 0.000 | 0.000 |
|  | FAERS | 0.000 | 0.000 | 0.000 | 0.000 | 0.000 | 0.000 |
|  | Florida | 0.000 | -0.001 | 0.000 | 0.000 | 0.000 | 0.000 |
|  | MIMIC-III | -0.015 | -0.017 | -0.013 | -0.012 | -0.013 | -0.011 |
|  | New York | 0.000 | 0.000 | 0.001 | 0.001 | 0.000 | 0.001 |
|  | NEXOID | 0.000 | 0.000 | 0.000 | 0.000 | 0.000 | 0.000 |
|  | Texas | 0.000 | 0.000 | 0.000 | 0.000 | 0.000 | 0.000 |
|  | Washington | 0.000 | -0.001 | 0.000 | 0.000 | 0.000 | 0.000 |
|  | Washington 2008 | -0.001 | -0.001 | 0.000 | 0.000 | -0.001 | 0.000 |
| TVAE | BORN | 0.000 | 0.000 | 0.000 | 0.000 | 0.000 | 0.000 |
|  | California | 0.000 | 0.000 | 0.000 | 0.000 | 0.000 | 0.000 |
|  | CCHS | 0.000 | -0.002 | 0.000 | 0.000 | 0.000 | 0.000 |
|  | COVID-19 | 0.000 | 0.000 | 0.000 | 0.000 | 0.000 | 0.000 |
|  | FAERS | 0.000 | 0.000 | 0.000 | 0.000 | 0.000 | 0.000 |
|  | Florida | 0.000 | 0.000 | 0.000 | 0.000 | 0.000 | 0.000 |
|  | MIMIC-III | -0.017 | -0.019 | -0.015 | -0.015 | -0.016 | -0.015 |
|  | New York | 0.001 | 0.000 | 0.001 | 0.001 | 0.001 | 0.001 |
|  | NEXOID | 0.000 | 0.000 | 0.001 | 0.000 | 0.000 | 0.000 |
|  | Texas | 0.000 | 0.000 | 0.000 | 0.000 | 0.000 | 0.000 |
|  | Washington | 0.000 | -0.001 | 0.000 | 0.000 | 0.000 | 0.000 |
|  | Washington 2008 | 0.000 | 0.000 | 0.000 | 0.000 | 0.000 | 0.000 |
| RTVAE | BORN | 0.000 | 0.000 | 0.000 | 0.000 | 0.000 | 0.000 |
|  | California | 0.000 | 0.000 | 0.000 | 0.000 | 0.000 | 0.000 |
|  | CCHS | -0.001 | -0.004 | 0.000 | 0.000 | 0.000 | 0.000 |
|  | COVID-19 | -0.001 | -0.002 | 0.000 | 0.000 | 0.000 | 0.000 |
|  | FAERS | 0.000 | 0.000 | 0.000 | 0.000 | 0.000 | 0.000 |
|  | Florida | -0.004 | -0.005 | -0.004 | -0.004 | -0.004 | -0.004 |
|  | MIMIC-III | -0.641 | -0.643 | -0.458 | -0.639 | -0.640 | -0.386 |
|  | New York | -0.004 | -0.007 | -0.004 | -0.004 | -0.004 | -0.003 |
|  | NEXOID | 0.000 | -0.005 | 0.000 | 0.000 | 0.000 | 0.000 |
|  | Texas | 0.000 | 0.000 | 0.000 | 0.000 | 0.000 | 0.000 |
|  | Washington | -0.020 | -0.021 | -0.020 | -0.020 | -0.020 | -0.020 |
|  | Washington 2008 | -0.022 | -0.023 | -0.022 | -0.022 | -0.022 | -0.022 |
| NFlow | BORN | 0.000 | 0.000 | 0.000 | 0.000 | 0.000 | 0.000 |
|  | California | 0.000 | 0.000 | 0.000 | 0.000 | 0.000 | 0.000 |
|  | CCHS | -0.003 | -0.016 | 0.000 | 0.000 | 0.000 | 0.000 |
|  | COVID-19 | 0.000 | -0.001 | 0.000 | 0.000 | 0.000 | 0.000 |
|  | FAERS | 0.000 | 0.000 | 0.000 | 0.000 | 0.000 | 0.000 |
|  | Florida | 0.000 | 0.000 | 0.000 | 0.000 | 0.000 | 0.000 |
|  | MIMIC-III | -0.010 | -0.014 | -0.008 | -0.009 | -0.010 | -0.006 |
|  | New York | 0.000 | -0.001 | 0.001 | 0.001 | 0.000 | 0.001 |
|  | NEXOID | 0.000 | 0.000 | 0.000 | 0.000 | 0.000 | 0.000 |
|  | Texas | 0.000 | 0.000 | 0.000 | 0.000 | 0.000 | 0.000 |
|  | Washington | 0.000 | 0.000 | 0.000 | 0.000 | 0.000 | 0.000 |
|  | Washington 2008 | -0.001 | -0.002 | 0.000 | 0.000 | -0.001 | 0.000 |

**Table 20.** **Results for Membership Disclosure Vulnerability Across Variants per SDG Model and Medical Dataset.** Privacy was assessed as membership disclosure vulnerability. The distribution across variants per medical dataset is reported as median, 1^st^ (Q1) and 3^rd^ quartile (Q3) for the synthetic subset (i.e., core) as well as the synthetic dataset with *core* and *adjunct* variables.


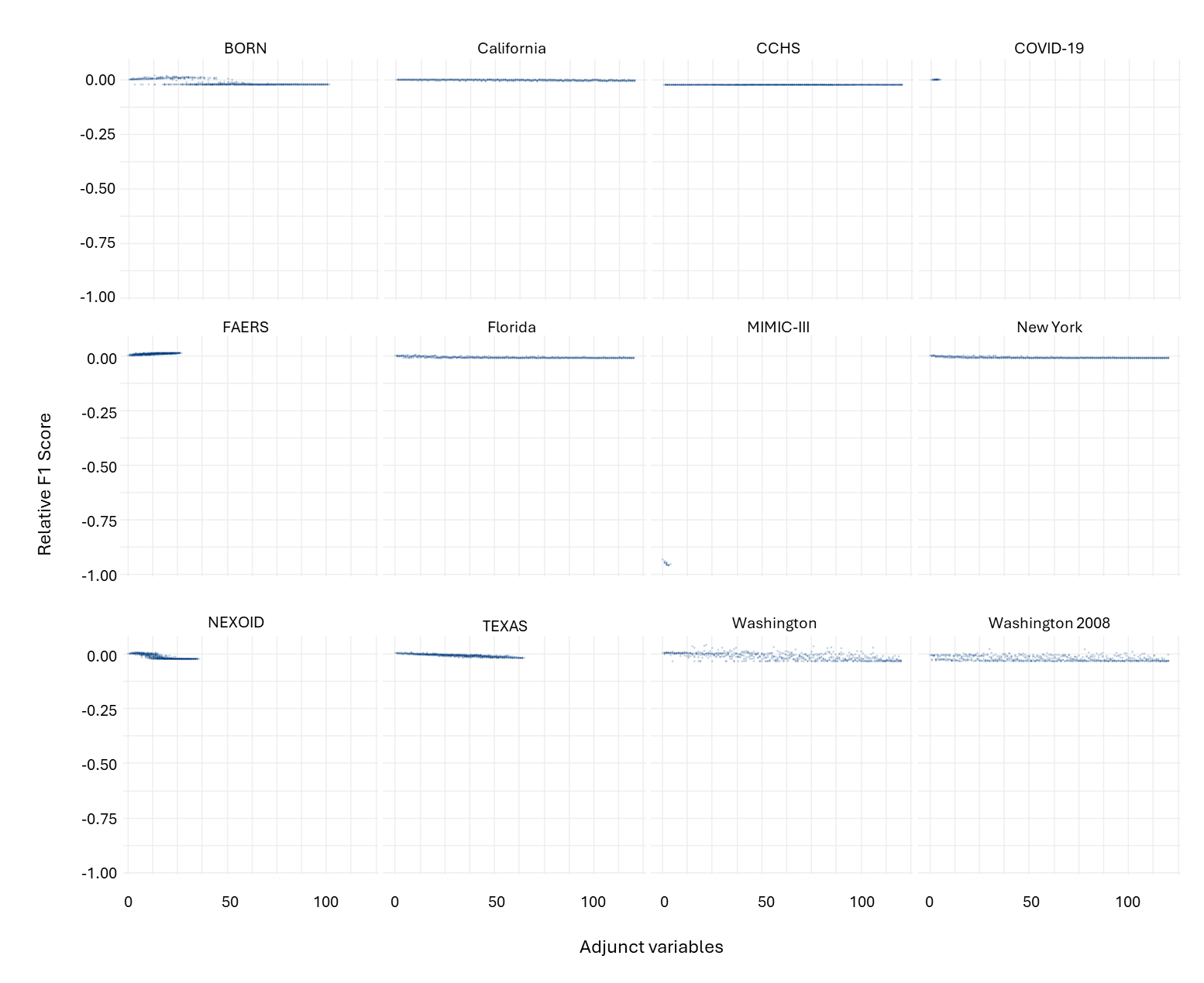


**Figure 4. Membership Disclosure Vulnerability Based on All Variables for the SDG Model ST.**  Membership disclosure vulnerability was measured for the entire synthetic dataset using all variables and the relative F1 score is indicated. The values were averaged across the 10 synthetic datasets per trained SDG model. Negative values indicate that the adversary’s success is worse than a naïve guess.


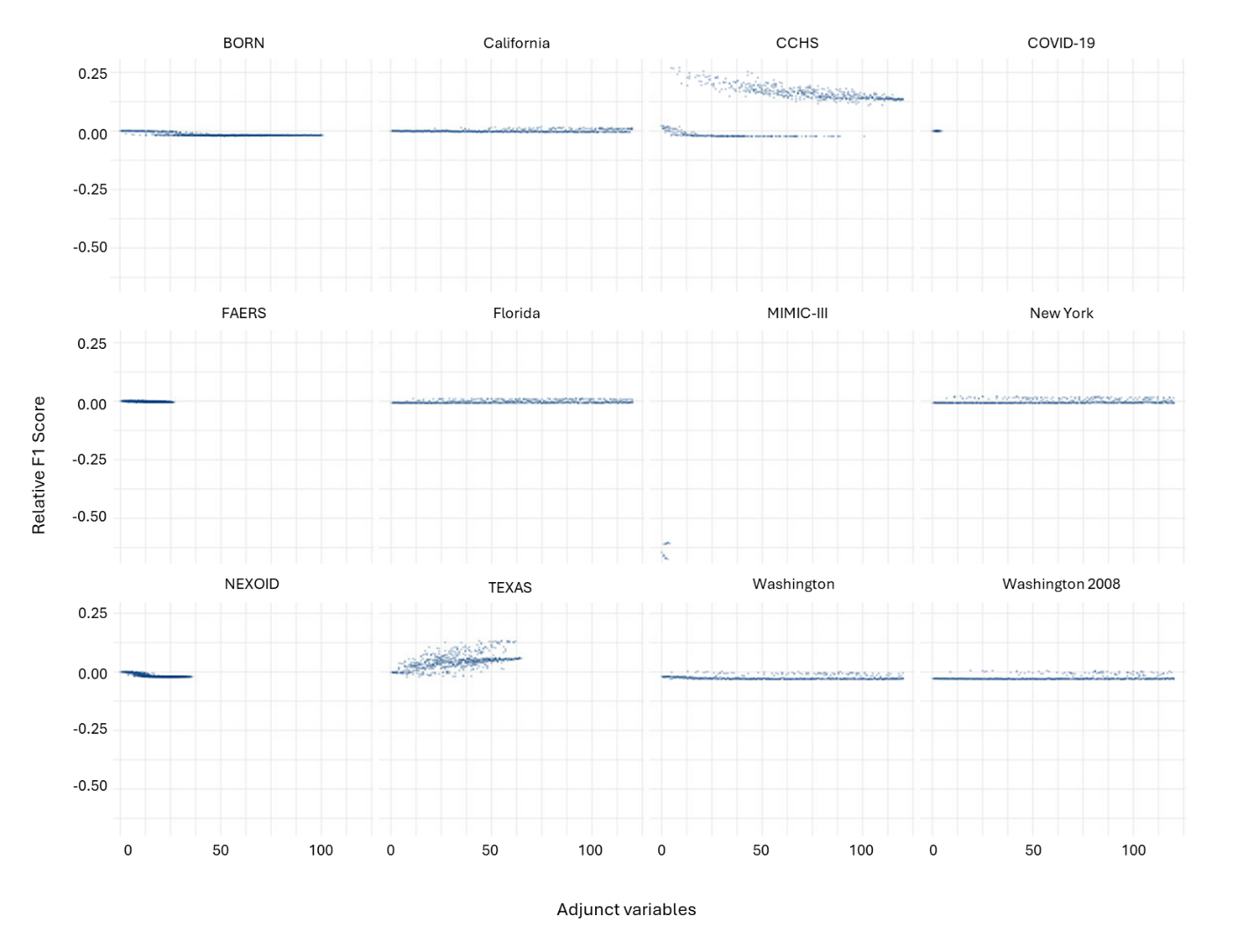


**Figure 5. Membership Disclosure Vulnerability Based on All Variables for the SDG Model BN.**  Membership disclosure vulnerability was measured for the entire synthetic dataset using all variables and the relative F1 score is indicated. The values were averaged across the 10 synthetic datasets per trained SDG model. Negative values indicate that the adversary’s success is worse than a naïve guess.


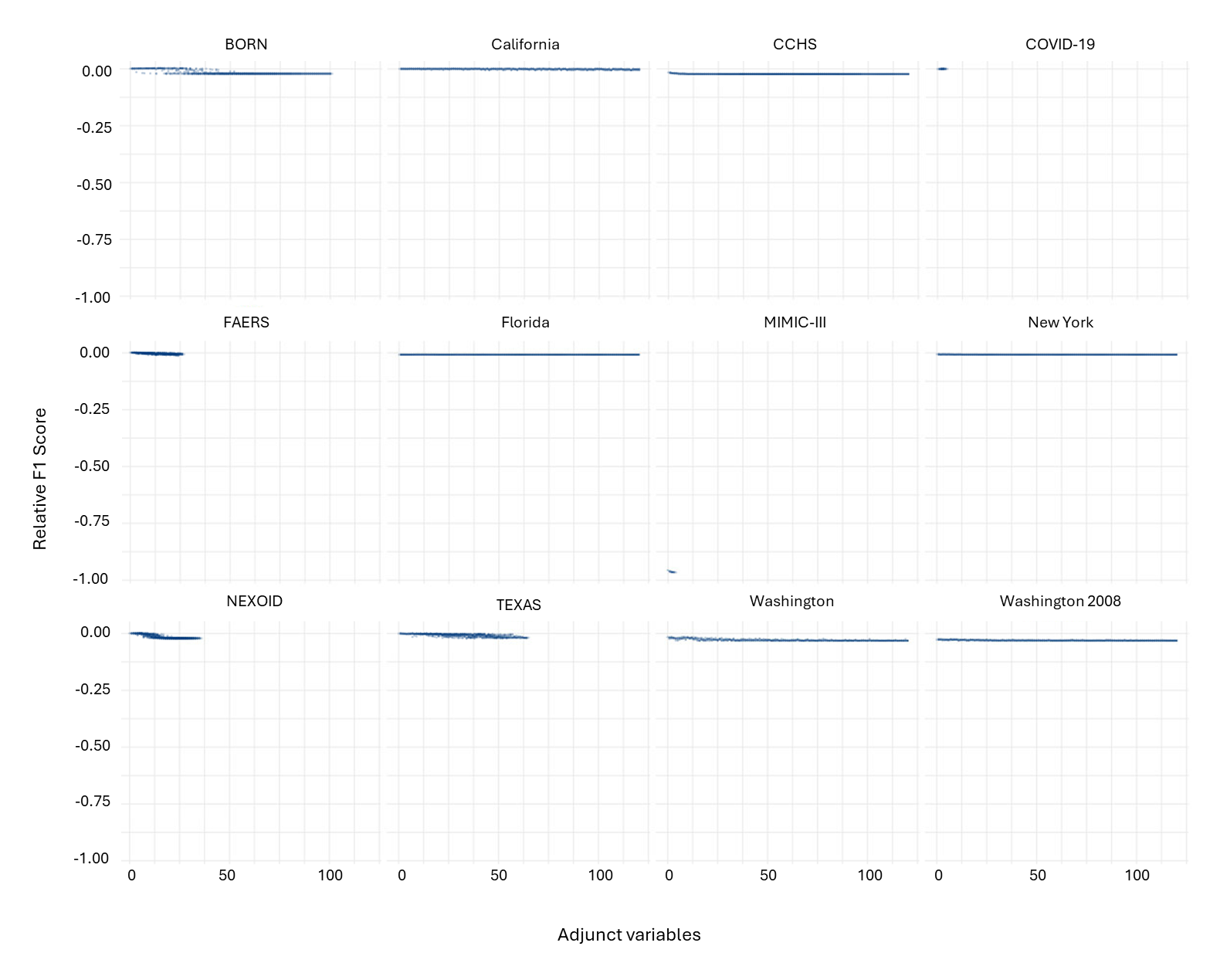


**Figure 6. Membership Disclosure Vulnerability Based on All Variables for the SDG Model ARF.**  Membership disclosure vulnerability was measured for the entire synthetic dataset using all variables and the relative F1 score is indicated. The values were averaged across the 10 synthetic datasets per trained SDG model. Negative values indicate that the adversary’s success is worse than a naïve guess.


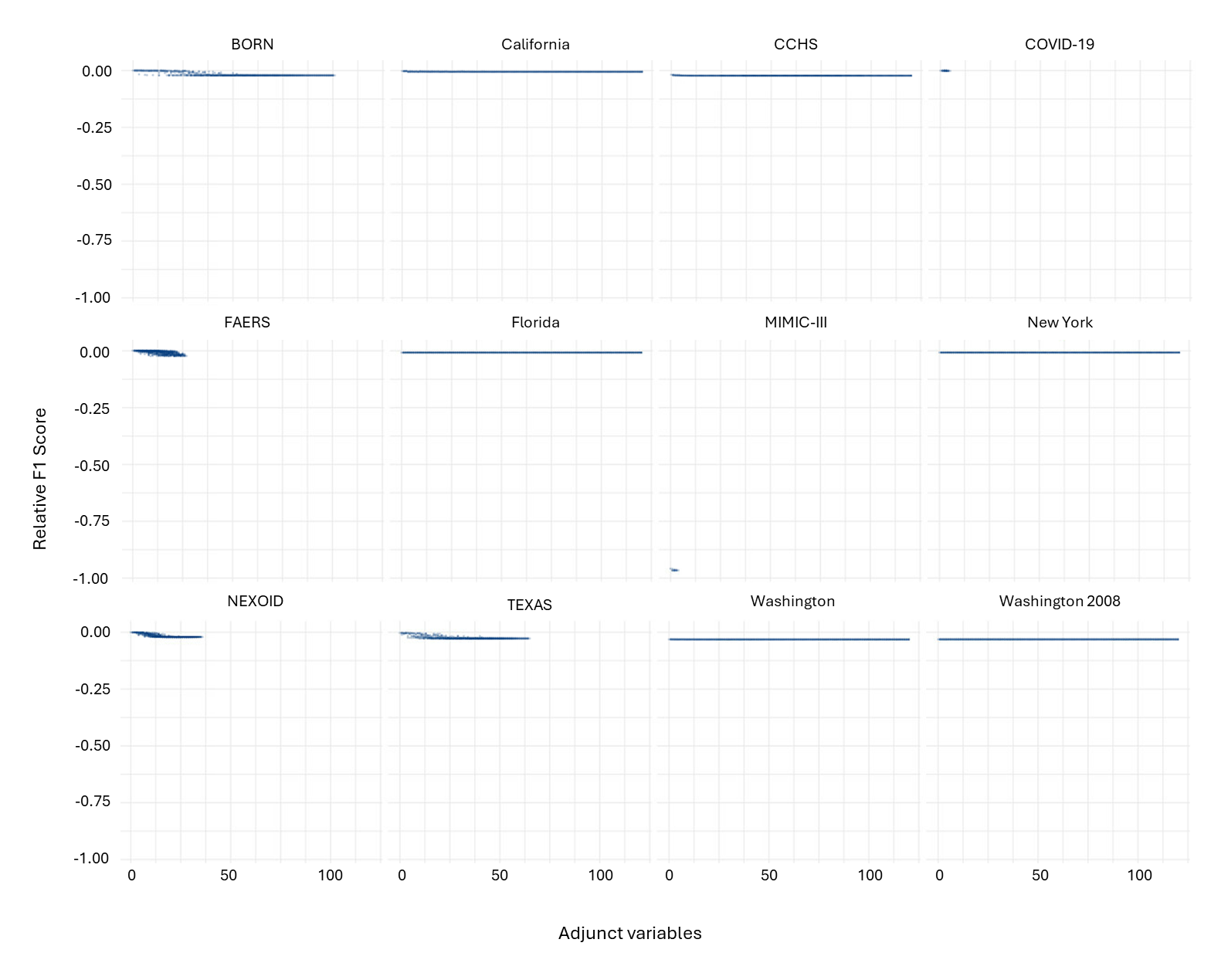


**Figure 7. Membership Disclosure Vulnerability Based on All Variables for the SDG Model CTGAN.**  Membership disclosure vulnerability was measured for the entire synthetic dataset using all variables and the relative F1 score is indicated. The values were averaged across the 10 synthetic datasets per trained SDG model. Negative values indicate that the adversary’s success is worse than a naïve guess.


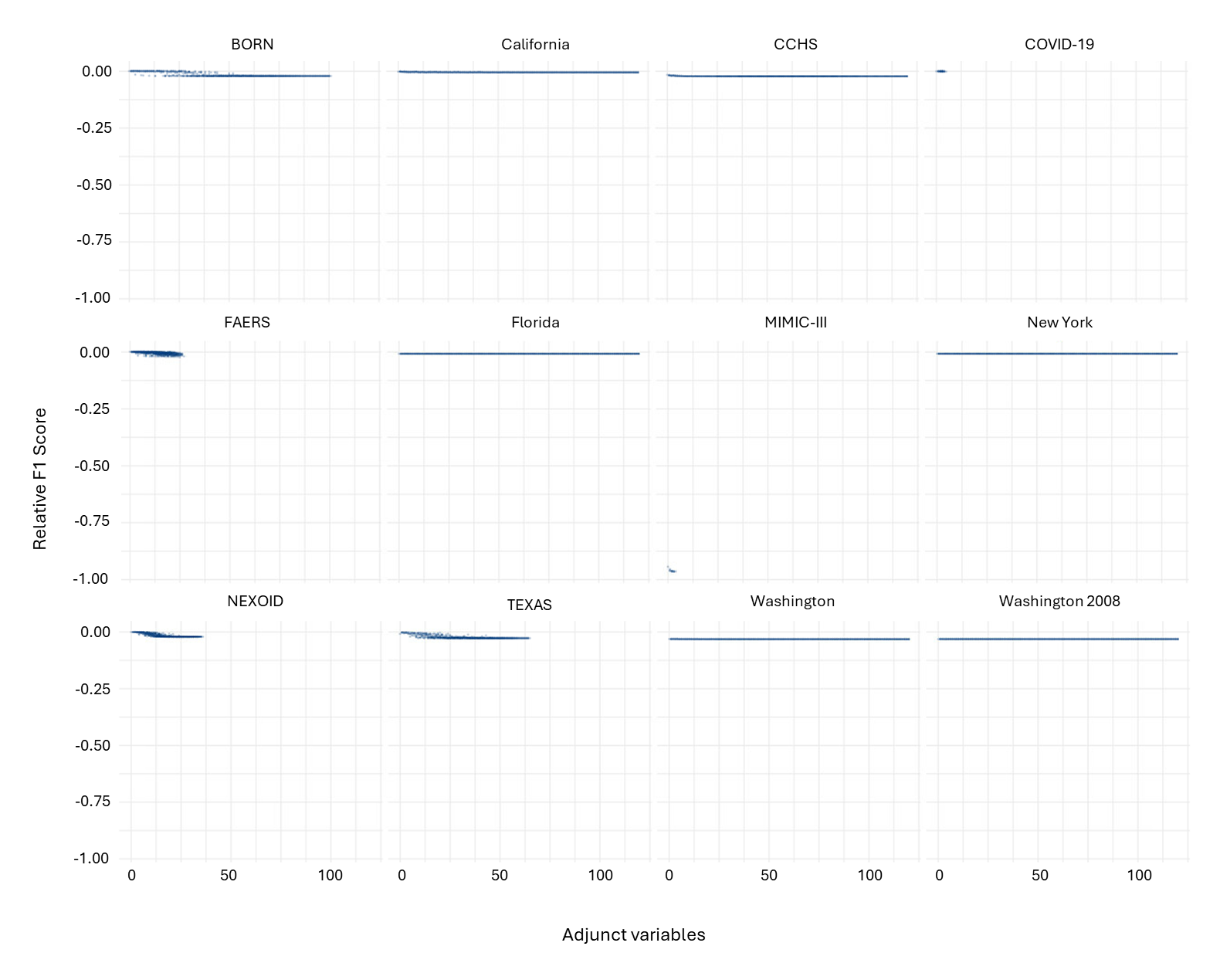


**Figure 8. Membership Disclosure Vulnerability Based on All Variables for the SDG Model TVAE.**  Membership disclosure vulnerability was measured for the entire synthetic dataset using all variables and the relative F1 score is indicated. The values were averaged across the 10 synthetic datasets per trained SDG model. Negative values indicate that the adversary’s success is worse than a naïve guess.


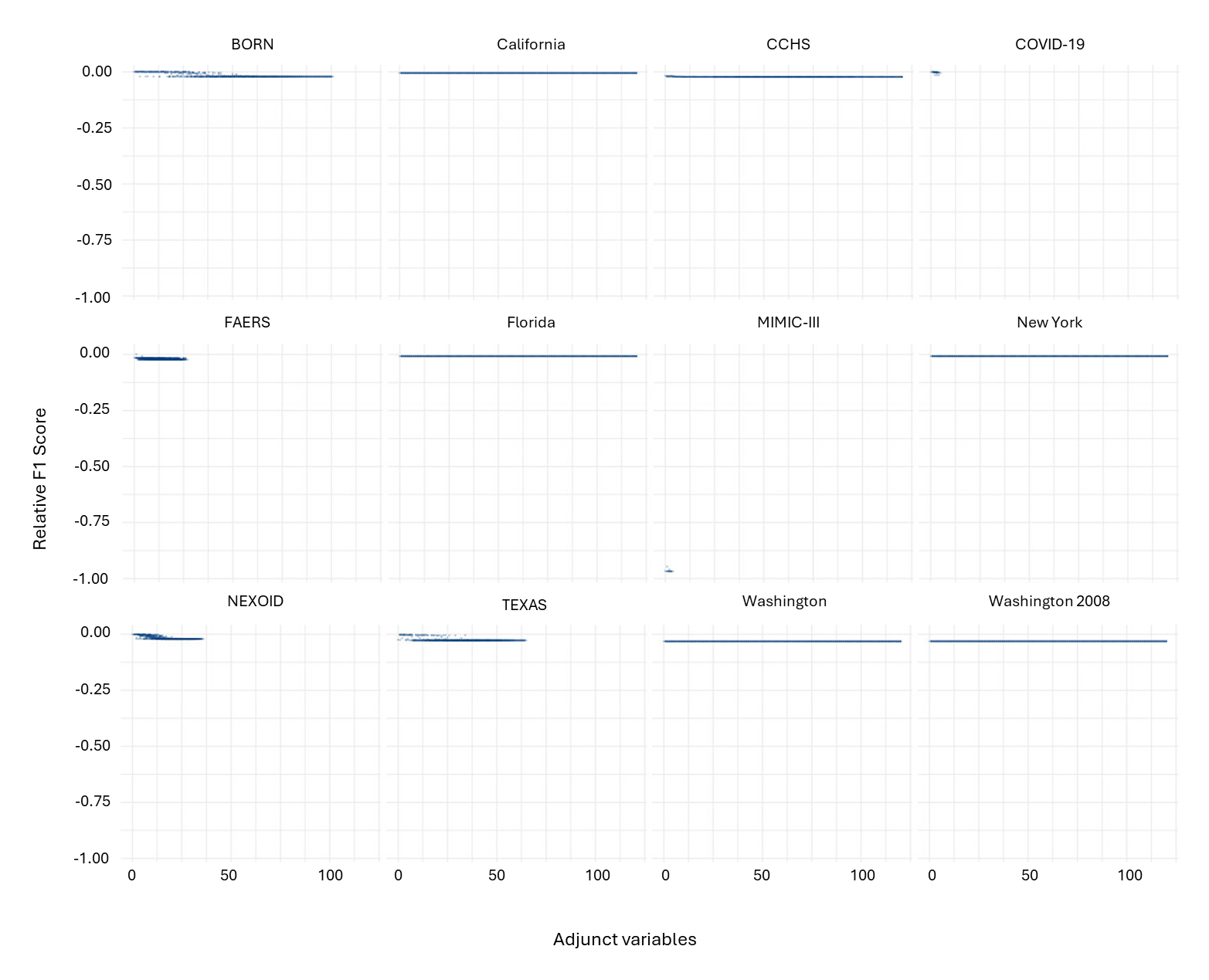


**Figure 9. Membership Disclosure Vulnerability Based on All Variables for the SDG Model RTVAE.**  Membership disclosure vulnerability was measured for the entire synthetic dataset using all variables and the relative F1 score is indicated. The values were averaged across the 10 synthetic datasets per trained SDG model. Negative values indicate that the adversary’s success is worse than a naïve guess.


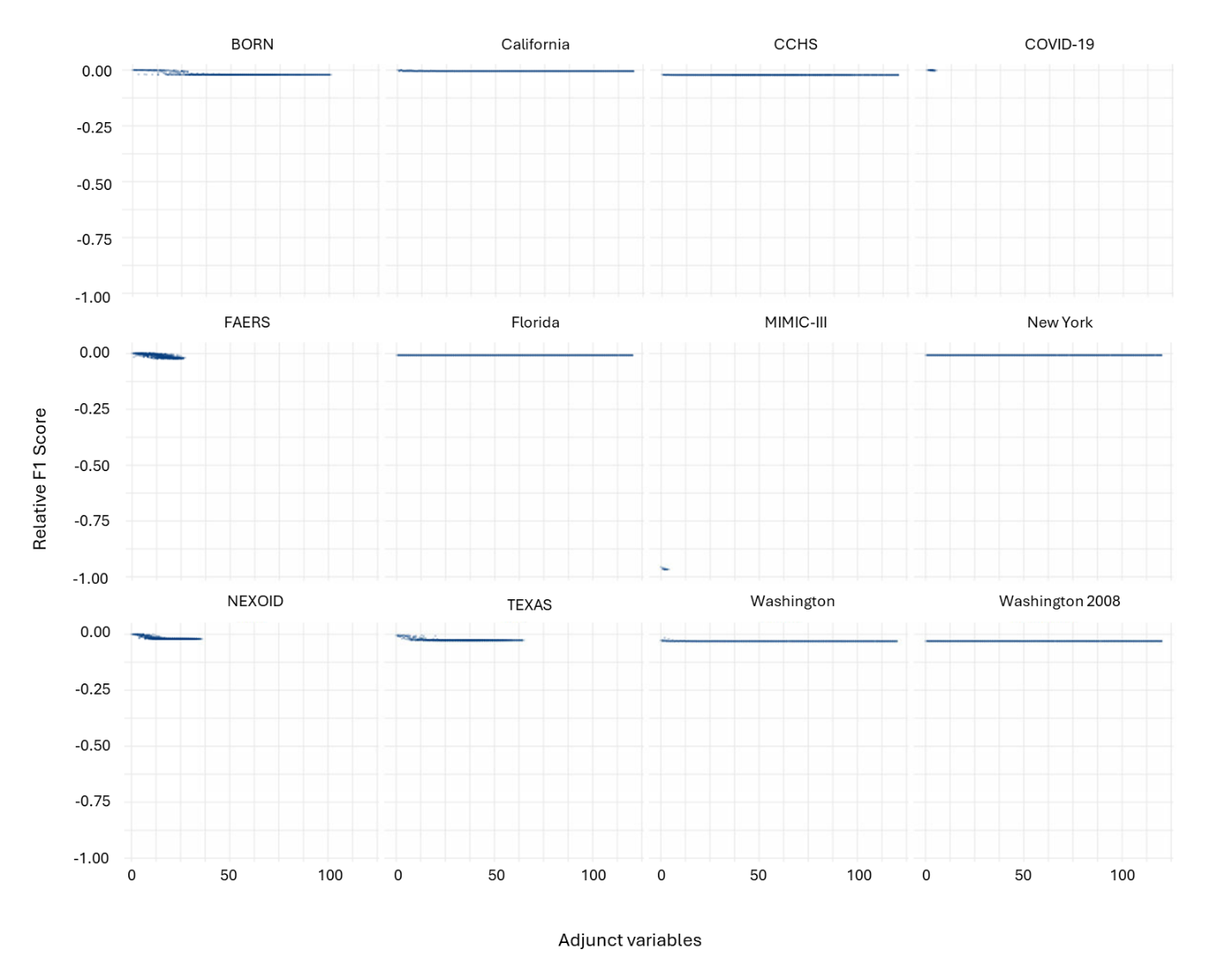


**Figure 10. Membership Disclosure Vulnerability Based on All Variables for the SDG Model NFlow.**  Membership disclosure vulnerability was measured for the entire synthetic dataset using all variables and the relative F1 score is indicated. The values were averaged across the 10 synthetic datasets per trained SDG model. Negative values indicate that the adversary’s success is worse than a naïve guess.

# MUTUAL INFORMATION ANALYSIS ACROSS VARIABLES

To explore potential explanations for our main findings, we assessed how much a *core* variable’s information could be explained by another variable in the dataset. This analysis provides additional insights into why fidelity and utility were unaffected by the inclusion of more adjunct variables. The pairwise normalized mutual information (NMI) was assessed between each *core* variable and all other *core* variables and all potential *adjunct* variables from the entire variable pool of the respective dataset [85]. We normalized by the entropy of the respective core variable to allow for comparison which gives a value between 0 (no shared information) and 1 (complete information overlap). The median and IQR were calculated across all *core*-to-*core* pairs and *core*-to-*adjunct* pairs per dataset. For implementation, the library infotheo was leveraged [86].

There was no consistent trend when comparing the median NMI of *core* variables with other *core* variables with the one with *adjunct* variables. The distribution of the NMI per dataset is shown in **Figure 11**.


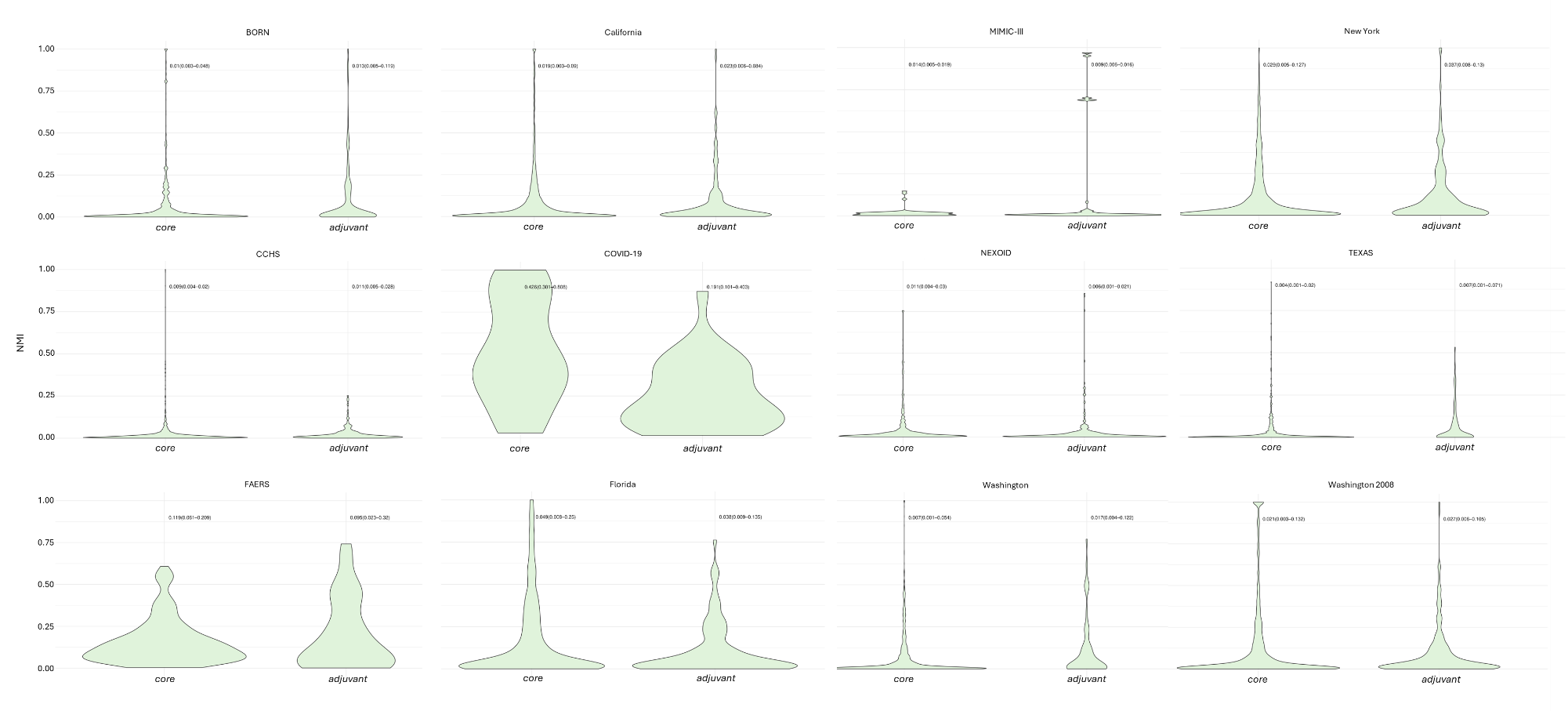


**Figure 11. Mutual Information Between *Core* Variables and Other *Core* and *Adjunct* Variables**. Mutual information was calculated between each *core*-to-*core* and *core*-to-*adjunct* variable pair, and normalized by the respective *core* variable’s entropy. All potential *adjunct* variables within the variable pool of each dataset were considered. The median across all other *core* and *adjunct* variables with its IQR is indicated.

# REFERENCES

[1] J. Valero De Bernabé *et al.*, “Risk factors for low birth weight: a review,” *Eur J Obstet Gynecol Reprod Biol*, vol. 116, no. 1, pp. 3–15, Sep. 2004, doi: 10.1016/j.ejogrb.2004.03.007.

[2] D. K. Yadav, U. Chaudhary, and N. Shrestha, “Risk factors associated with low birth weight,” *J Nepal Health Res Counc*, vol. 9, no. 2, pp. 159–164, Oct. 2011.

[3] “HCUP State Inpatient Databases (SID). Healthcare Cost and Utilization Project (HCUP). 2005-2009. Agency for Healthcare Research and Quality, Rockville, MD. www.hcup-us.ahrq.gov/sidoverview.jsp.”

[4] F. Ul and M. Ml, “Frequency, trends, and antecedents of severe maternal depression after three million U.S. births,” *PloS one*, vol. 13, no. 2, Feb. 2018, doi: 10.1371/journal.pone.0192854.

[5] S. A. Brownlee *et al.*, “Impact of Post-Hospital Syndrome on Outcomes Following Elective, Ambulatory Surgery,” *Ann Surg*, vol. 266, no. 2, pp. 274–279, Aug. 2017, doi: 10.1097/SLA.0000000000001965.

[6] L. C. Maclagan *et al.*, “The CANHEART health index: a tool for monitoring the cardiovascular health of the Canadian population,” *CMAJ*, vol. 186, no. 3, pp. 180–187, Feb. 2014, doi: 10.1503/cmaj.131358.

[7] I. Berry *et al.*, “A sub-national real-time epidemiological and vaccination database for the COVID-19 pandemic in Canada,” *Sci Data*, vol. 8, no. 1, p. 173, Jul. 2021, doi: 10.1038/s41597-021-00955-2.

[8] K. Marwitz, S. C. Jones, C. M. Kortepeter, G. J. Dal Pan, and M. A. Muñoz, “An Evaluation of Postmarketing Reports with an Outcome of Death in the US FDA Adverse Event Reporting System,” *Drug Saf*, vol. 43, no. 5, pp. 457–465, May 2020, doi: 10.1007/s40264-020-00908-5.

[9] J. Meddings *et al.*, “The Impact of Disability and Social Determinants of Health on Condition-Specific Readmissions beyond Medicare Risk Adjustments: A Cohort Study,” *J Gen Intern Med*, vol. 32, no. 1, pp. 71–80, Jan. 2017, doi: 10.1007/s11606-016-3869-x.

[10] A. Johnson, T. Pollard, and R. Mark, “MIMIC-III Clinical Database (version 1.4).” PhysioNet, PhysioNet, PhysioNet 2016. doi: https://doi.org/10.13026/C2XW26.

[11] A. E. W. Johnson *et al.*, “MIMIC-III, a freely accessible critical care database,” *Sci Data*, vol. 3, no. 1, Art. no. 1, May 2016, doi: 10.1038/sdata.2016.35.

[12] A. L. Goldberger *et al.*, “PhysioBank, PhysioToolkit, and PhysioNet: Components of a New Research Resource for Complex Physiologic Signals,” *Circulation*, vol. 101, no. 23, Jun. 2000, doi: 10.1161/01.CIR.101.23.e215.

[13] M. Pishgar, J. Theis, M. Del Rios, A. Ardati, H. Anahideh, and H. Darabi, “Prediction of unplanned 30-day readmission for ICU patients with heart failure,” *BMC Med Inform Decis Mak*, vol. 22, no. 1, p. 117, May 2022, doi: 10.1186/s12911-022-01857-y.

[14] O. Aliu *et al.*, “The effect of pre-Affordable Care Act (ACA) Medicaid eligibility expansion in New York State on access to specialty surgical care,” *Med Care*, vol. 52, no. 9, pp. 790–795, Sep. 2014, doi: 10.1097/MLR.0000000000000175.

[15] J. M. Kahn *et al.*, “The Epidemiology of Chronic Critical Illness in the United States,” *Crit Care Med*, vol. 43, no. 2, pp. 282–287, Feb. 2015, doi: 10.1097/CCM.0000000000000710.

[16] A. K. Sabbatini, K. E. Kocher, A. Basu, and R. Y. Hsia, “In-Hospital Outcomes and Costs Among Patients Hospitalized During a Return Visit to the Emergency Department,” *JAMA*, vol. 315, no. 7, pp. 663–671, Feb. 2016, doi: 10.1001/jama.2016.0649.

[17] J. Grantham, “COVID-19 Survival Calculator,” Nexoid’s COVID-19 Survival Calculator. Accessed: Dec. 22, 2020. [Online]. Available: https://www.covid19survivalcalculator.com

[18] “Texas Hospital Inpatient Discharge Public Use Data File, First Quarter, 2012, 1st quarter 2012. Texas Department of State Health Services, Center for Health Statistics, Austin, Texas.”

[19] J. Zhang and P. Yu, “Machine Learning Methods for Prediction of COVID-19 Patient Length of Stay: Using Texas PUDF Data,” in *2023 3rd International Conference on Electrical, Computer, Communications and Mechatronics Engineering (ICECCME)*, Jul. 2023, pp. 1–7. doi: 10.1109/ICECCME57830.2023.10252792.

[20] L. B. Goss, J. R. Ortiz, D. M. Okamura, K. Hayward, and C. H. Goss, “Significant Reductions in Mortality in Hospitalized Patients with Systemic Lupus Erythematosus in Washington State from 2003 to 2011,” *PLoS One*, vol. 10, no. 6, p. e0128920, 2015, doi: 10.1371/journal.pone.0128920.

[21] D. Metcalfe, C. K. Zogg, E. R. Haut, T. M. Pawlik, A. H. Haider, and D. C. Perry, “Data resource profile: State Inpatient Databases,” *International Journal of Epidemiology*, vol. 48, no. 6, pp. 1742–1742h, Dec. 2019, doi: 10.1093/ije/dyz117.

[22] M. L. Barrett, L. M. Wier, H. J. Jiang, and C. A. Steiner, “All-Cause Readmissions by Payer and Age, 2009–2013,” in *Healthcare Cost and Utilization Project (HCUP) Statistical Briefs [Internet]*, Agency for Healthcare Research and Quality (US), 2015. Accessed: Oct. 14, 2024. [Online]. Available: https://www.ncbi.nlm.nih.gov/books/NBK343800/

[23] K. El Emam, L. Mosquera, and C. Zheng, “Optimizing the synthesis of clinical trial data using sequential trees,” *J Am Med Inform Assoc*, Nov. 2020, doi: 10.1093/jamia/ocaa249.

[24] J. Drechsler and J. P. Reiter, “An empirical evaluation of easily implemented, nonparametric methods for generating synthetic datasets,” *Computational Statistics & Data Analysis*, vol. 55, no. 12, pp. 3232–3243, Dec. 2011, doi: 10.1016/j.csda.2011.06.006.

[25] B. Nowok, “Utility of synthetic microdata generated using tree-based methods,” presented at the UNECE Statistical Data Confidentiality Work Session, Helsinki, Oct. 2015. doi: https://unece.org/statistics/events/SDC2015.

[26] J. Reiter, “Using CART to generate partially synthetic, public use microdata,” *Journal of Official Statistics*, vol. 21, no. 3, pp. 441–462, 2005.

[27] D. Kaur *et al.*, “Application of Bayesian networks to generate synthetic health data,” *J Am Med Inform Assoc*, vol. 28, no. 4, pp. 801–811, Mar. 2021, doi: 10.1093/jamia/ocaa303.

[28] G. Gogoshin, S. Branciamore, and A. S. Rodin, “Synthetic data generation with probabilistic Bayesian Networks,” *Math Biosci Eng*, vol. 18, no. 6, pp. 8603–8621, Oct. 2021, doi: 10.3934/mbe.2021426.

[29] L. N. A. Martins, F. B. Gonçalves, and T. P. Galletti, “Generation and analysis of synthetic data via Bayesian networks: a robust approach for uncertainty quantification via Bayesian paradigm,” Feb. 29, 2024, *arXiv*: arXiv:2402.17915. doi: 10.48550/arXiv.2402.17915.

[30] I. Deeva, P. D. Andriushchenko, A. V. Kalyuzhnaya, and A. V. Boukhanovsky, “Bayesian Networks-based personal data synthesis,” in *Proceedings of the 6th EAI International Conference on Smart Objects and Technologies for Social Good*, in GoodTechs ’20. New York, NY, USA: Association for Computing Machinery, Sep. 2020, pp. 6–11. doi: 10.1145/3411170.3411243.

[31] L. Xu, M. Skoularidou, A. Cuesta-Infante, and K. Veeramachaneni, “Modeling Tabular data using Conditional GAN,” in *Advances in Neural Information Processing Systems*, vol. 32, 2019. [Online]. Available: https://papers.nips.cc/paper/2019/hash/254ed7d2de3b23ab10936522dd547b78-Abstract.html

[32] D. S. Watson, K. Blesch, J. Kapar, and M. N. Wright, “Adversarial random forests for density estimation and generative modeling,” Mar. 13, 2023, *arXiv*: arXiv:2205.09435. doi: 10.48550/arXiv.2205.09435.

[33] C. Durkan, A. Bekasov, I. Murray, and G. Papamakarios, “Neural Spline Flows,” Dec. 02, 2019, *arXiv*: arXiv:1906.04032. doi: 10.48550/arXiv.1906.04032.

[34] Z. Qian, B.-C. Cebere, and M. van der Schaar, “Synthcity: facilitating innovative use cases of synthetic data in different data modalities,” *arXiv*, vol. 2301.07573, Jan. 2023, Accessed: Oct. 10, 2023. [Online]. Available: https://arxiv.org/abs/2301.07573v1

[35] K. E. Emam, S. E. Kababji, L. Pilgram, V. Cano, and D. Liu, “pysdg,” Jul. 2024, doi: 10.17605/OSF.IO/XJ9PR.

[36] T. Hothorn, K. Hornik, and A. Zeileis, “Unbiased Recursive Partitioning: A Conditional Inference Framework,” *Journal of Computational and Graphical Statistics*, vol. 15, no. 3, pp. 651–674, Sep. 2006, doi: 10.1198/106186006X133933.

[37] J. Read, B. Pfahringer, G. Holmes, and E. Frank, “Classifier Chains for Multi-label Classification,” in *Machine Learning and Knowledge Discovery in Databases*, W. Buntine, M. Grobelnik, D. Mladenić, and J. Shawe-Taylor, Eds., in Lecture Notes in Computer Science. Berlin, Heidelberg: Springer, 2009, pp. 254–269. doi: 10.1007/978-3-642-04174-7_17.

[38] R. C. Arslan, K. M. Schilling, T. M. Gerlach, and L. Penke, “Using 26,000 diary entries to show ovulatory changes in sexual desire and behavior,” *J Pers Soc Psychol*, vol. 121, no. 2, pp. 410–431, 2021, doi: 10.1037/pspp0000208.

[39] D. Bonnéry *et al.*, “The Promise and Limitations of Synthetic Data as a Strategy to Expand Access to State-Level Multi-Agency Longitudinal Data,” *Journal of Research on Educational Effectiveness*, vol. 12, no. 4, pp. 616–647, Oct. 2019, doi: 10.1080/19345747.2019.1631421.

[40] A. Sabay, L. Harris, V. Bejugama, and K. Jaceldo-Siegl, “Overcoming Small Data Limitations in Heart Disease Prediction by Using Surrogate Data,” *SMU Data Science Review*, vol. 1, no. 3, p. Article 12, Aug. 2018.

[41] Michael Freiman, Amy Lauger, and Jerome Reiter, “Data Synthesis and Perturbation for the American Community Survey at the U.S. Census Bureau,” US Census Bureau. https://www.census.gov/library/working-papers/2018/adrm/formal-privacy-synthetic-data-acs.html, Working paper, 2017. Accessed: Feb. 24, 2020. [Online]. Available: https://www.census.gov/library/working-papers/2018/adrm/formal-privacy-synthetic-data-acs.html

[42] G. M. Raab, B. Nowok, and C. Dibben, “Practical Data Synthesis for Large Samples,” *Journal of Privacy and Confidentiality*, vol. 7, no. 3, pp. 67–97, 2016, doi: 10.29012/jpc.v7i3.407.

[43] B. Nowok, G. M. Raab, and C. Dibben, “Providing bespoke synthetic data for the UK Longitudinal Studies and other sensitive data with the synthpop package for R 1,” *Statistical Journal of the IAOS*, vol. 33, no. 3, pp. 785–796, Jan. 2017, doi: 10.3233/SJI-150153.

[44] D. S. Quintana, “A synthetic dataset primer for the biobehavioural sciences to promote reproducibility and hypothesis generation,” *eLife*, vol. 9, p. e53275, 2020, doi: 10.7554/eLife.53275.

[45] K. P. Murphy, *Machine Learning: A Probabilistic Perspective*. MIT Press, 2012.

[46] Z. Qian, B.-C. Cebere, and M. van der Schaar, “Synthcity: facilitating innovative use cases of synthetic data in different data modalities.” 2023.

[47] I. Goodfellow *et al.*, “Generative adversarial nets,” in *Advances in neural information processing systems*, 2014, pp. 2672–2680.

[48] S. Bourou, A. El Saer, T. Velivasaki, A. Voulkidis, and T. Zahariadis, “A Review of Tabular Data Synthesis Using GANs on an IDS Dataset,” *Information*, vol. 12, p. 375, Sep. 2021, doi: 10.3390/info12090375.

[49] D. P. Kingma and M. Welling, “Auto-Encoding Variational Bayes,” Dec. 2013. doi: 10.48550/arXiv.1312.6114.

[50] Z. Wan, Y. Zhang, and H. He, “Variational autoencoder based synthetic data generation for imbalanced learning,” in *2017 IEEE Symposium Series on Computational Intelligence (SSCI)*, Nov. 2017, pp. 1–7. doi: 10.1109/SSCI.2017.8285168.

[51] H. Ishfaq, A. Hoogi, and D. Rubin, “TVAE: Triplet-Based Variational Autoencoder using Metric Learning,” Feb. 08, 2023, *arXiv*: arXiv:1802.04403. doi: 10.48550/arXiv.1802.04403.

[52] K. Sohn, H. Lee, and X. Yan, “Learning Structured Output Representation using Deep Conditional Generative Models,” in *Advances in Neural Information Processing Systems*, Curran Associates, Inc., 2015. Accessed: Aug. 14, 2024. [Online]. Available: https://papers.nips.cc/paper/2015/hash/8d55a249e6baa5c06772297520da2051-Abstract.html

[53] A. Salim, *Synthetic Patient Generation: A Deep Learning Approach Using Variational Autoencoders*. 2018.

[54] H. Akrami, A. A. Joshi, J. Li, S. Aydöre, and R. M. Leahy, “A robust variational autoencoder using beta divergence,” *Knowledge-Based Systems*, vol. 238, p. 107886, Feb. 2022, doi: 10.1016/j.knosys.2021.107886.

[55] A. Goncalves, P. Ray, B. Soper, J. Stevens, L. Coyle, and A. P. Sales, “Generation and evaluation of synthetic patient data,” *BMC Medical Research Methodology*, vol. 20, no. 1, p. 108, May 2020, doi: 10.1186/s12874-020-00977-1.

[56] M.-J. Woo, J. P. Reiter, A. Oganian, and A. F. Karr, “Global Measures of Data Utility for Microdata Masked for Disclosure Limitation,” *Journal of Privacy and Confidentiality*, vol. 1, no. 1, Apr. 2009, doi: 10.29012/jpc.v1i1.568.

[57] R. Tibshirani, G. Walther, and T. Hastie, “Estimating the number of clusters in a data set via the gap statistic,” *Journal of the Royal Statistical Society: Series B (Statistical Methodology)*, vol. 63, no. 2, pp. 411–423, 2001, doi: 10.1111/1467-9868.00293.

[58] S. L. Hyland, C. Esteban, and G. Rätsch, “Real-valued (Medical) Time Series Generation with Recurrent Conditional GANs,” *arXiv:1706.02633 [cs, stat]*, Jun. 2017, Accessed: May 28, 2019. [Online]. Available: http://arxiv.org/abs/1706.02633

[59] P. K. Kushwaha and M. Kumaresan, “Machine learning algorithm in healthcare system: A Review,” in *2021 International Conference on Technological Advancements and Innovations (ICTAI)*, Nov. 2021, pp. 478–481. doi: 10.1109/ICTAI53825.2021.9673220.

[60] S. Gupta and R. R. Sedamkar, “Machine Learning for Healthcare: Introduction,” in *Machine Learning with Health Care Perspective: Machine Learning and Healthcare*, V. Jain and J. M. Chatterjee, Eds., Cham: Springer International Publishing, 2020, pp. 1–25. doi: 10.1007/978-3-030-40850-3_1.

[61] T. J. Bradshaw, Z. Huemann, J. Hu, and A. Rahmim, “A Guide to Cross-Validation for Artificial Intelligence in Medical Imaging,” *Radiology: Artificial Intelligence*, vol. 5, no. 4, p. e220232, Jul. 2023, doi: 10.1148/ryai.220232.

[62] E. Bartz, T. Bartz-Beielstein, M. Zaefferer, and O. Mersmann, Eds., *Hyperparameter Tuning for Machine and Deep Learning with R: A Practical Guide*. Singapore: Springer Nature, 2023. doi: 10.1007/978-981-19-5170-1.

[63] B. Bischl *et al.*, “Hyperparameter Optimization: Foundations, Algorithms, Best Practices and Open Challenges,” arXiv.org. Accessed: Dec. 09, 2023. [Online]. Available: https://arxiv.org/abs/2107.05847v3

[64] M. Binder, F. Pﬁsterer, and B. Bischl, “Collecting Empirical Data About Hyperparameters for Data Driven AutoML,” *7th ICML Workshop on Automated Machine Learning*, 2020.

[65] D. Kühn, P. Probst, J. Thomas, and B. Bischl, “Automatic Exploration of Machine Learning Experiments on OpenML,” arXiv.org. Accessed: Dec. 09, 2023. [Online]. Available: https://arxiv.org/abs/1806.10961v3

[66] K. E. Emam, L. Mosquera, and C. Zheng, “Optimizing the Synthesis of Clinical Trial Data Using Sequential Trees,” *Journal of the American Medical Informatics Association*, Nov. 2020, [Online]. Available: https://academic.oup.com/jamia/advance-article/doi/10.1093/jamia/ocaa249/5981525

[67] L. Juwara, A. El-Hussuna, and K. El Emam, “An evaluation of synthetic data augmentation for mitigating covariate bias in health data,” *Patterns*, 2024, doi: 10.1016/j.patter.2024.100946.

[68] Y. Huang, W. Li, F. Macheret, R. A. Gabriel, and L. Ohno-Machado, “A tutorial on calibration measurements and calibration models for clinical prediction models,” *J Am Med Inform Assoc*, vol. 27, no. 4, pp. 621–633, Apr. 2020, doi: 10.1093/jamia/ocz228.

[69] M. Kull, T. S. Filho, and P. Flach, “Beta calibration: a well-founded and easily implemented improvement on logistic calibration for binary classifiers,” in *Proceedings of the 20th International Conference on Artificial Intelligence and Statistics*, PMLR, Apr. 2017, pp. 623–631. doi: https://proceedings.mlr.press/v54/kull17a.html.

[70] K. E. Emam, “sdgm Package,” Jan. 2024, doi: 10.17605/OSF.IO/DCJM6.

[71] “TensorFlow for R - Reference.” Accessed: Jan. 22, 2025. [Online]. Available: https://tensorflow.rstudio.com/reference/

[72] K. El Emam, L. Mosquera, and X. Fang, “Validating A Membership Disclosure Metric For Synthetic Health Data,” *JAMIA Open*, vol. 5, no. 4, p. ooac083, Dec. 2022.

[73] K. El Emam, *Guide to the De-Identification of Personal Health Information*. Boca Raton, FL: CRC Press (Auerbach), 2013.

[74] International Standards Organization, “ISO/IEC 27559:2022: Information security, cybersecurity and privacy protection – Privacy enhancing data de-identification framework,” ISO, 2022.

[75] Article 29 Data Protection Working Party, “Opinion 05/2014 on Anonymization Techniques,” Apr. 2014.

[76] EMA, “External Guidance on the Implementation of the European Medicines Agency Policy on the Publication of Clinical Data for Medicinal Products for Human Use,” 2018.

[77] Anco Hundepool *et al.*, *Statistical Disclosure Control*. Wiley, 2012. Accessed: May 10, 2015. [Online]. Available: http://ca.wiley.com/WileyCDA/WileyTitle/productCd-1119978157.html

[78] G. T. Duncan, M. Elliot, and J.-J. Salazar-González, *Statistical Confidentiality*. New York, NY: Springer New York, 2011. Accessed: Aug. 10, 2015. [Online]. Available: http://link.springer.com/10.1007/978-1-4419-7802-8

[79] M. Templ, “Statistical Disclosure Control for Microdata: Methods and Applications in R.” Springer, 2017.

[80] L. Willenborg and T. de Waal, *Statistical Disclosure Control in Practice*. New York: Springer-Verlag, 1996.

[81] L. Willenborg and T. de Wall, *Elements of Statistical Disclosure Control*, 1st ed. in Lecture Notes in Statistics, no. 155. Springer-Verlag New York, 2001. Accessed: Nov. 21, 2017. [Online]. Available: DOI:10.1007/978-1-4613-0121-9

[82] M. Elliot and A. Dale, “Scenarios of Attack: The Data Intruders Perspective on Statistical Disclosure Risk,” *Netherlands Official Statistics*, vol. 14, no. Spring, pp. 6–10, 1999.

[83] L. Pilgram *et al.*, “A consensus privacy metrics framework for synthetic data,” *PATTER*, vol. 0, no. 0, Jul. 2025, doi: 10.1016/j.patter.2025.101320.

[84] H. Sun, T. Zhu, Z. Zhang, D. Jin, P. Xiong, and W. Zhou, “Adversarial Attacks Against Deep Generative Models on Data: A Survey,” *IEEE Transactions on Knowledge and Data Engineering*, no. 01, pp. 1–1, Nov. 2021, doi: 10.1109/TKDE.2021.3130903.

[85] “Entropy, Relative Entropy, and Mutual Information,” in *Elements of Information Theory*, John Wiley & Sons, Ltd, 2005, pp. 13–55. doi: 10.1002/047174882X.ch2.

[86] P. E. Meyer, *infotheo: Information-Theoretic Measures*. (Apr. 08, 2022). Accessed: Jun. 30, 2025. [Online]. Available: https://cran.r-project.org/web/packages/infotheo/index.html

1. See <<https://aetion.com/products/generate/>> [↑](#footnote-ref-2)
